# Exponential dissipative control for conformable nonlinear dynamical systems

**DOI:** 10.1038/s41598-026-44606-1

**Published:** 2026-04-30

**Authors:** Slim Dhahri, Essia Ben Alaia, Sahar Almenwer, Afrah Alanazi, Hamdi Gassara

**Affiliations:** 1 Department of Computer Engineering and Networks, College of Computer and Information Sciences, Jouf University, Sakaka, Saudi Arabia; 2 Department of Computer Science, College of Computer and Information Sciences, Jouf University, Sakaka, Saudi Arabia; 3 Department of Information System, College of Computer and Information Sciences, Jouf University, Sakaka, Saudi Arabia; 4 Laboratory of Sciences and Tech. of Automatic Control and Computer Eng., National School of Engineering of Sfax, PB 1173, 3038 Sfax, Tunisia

**Keywords:** Conformable fractional-order derivative, SOS approach, Exponential stability analysis, Observer design, Polynomial fuzzy model, Engineering, Mathematics and computing, Physics

## Abstract

This paper addresses the design of a controller that exponentially stabilizes Conformable Nonlinear Dynamical Systems (CNDSs) with time delay. To address the nonlinear dynamics of the system, we employ a Polynomial Fuzzy (PF) modeling approach. This method provides an accurate representation for a large class of CNDSs and serves as a generalized framework that extends the classical Takagi-Sugeno Fuzzy (TSF) model. The controller is designed to ensure not only exponential stability, which implies a prescribed convergence rate compared to asymptotic stability, but also the strictly $(\mathcal {U},\mathcal {V},\mathcal {W})$-$\sigma$-dissipativity of the closed-loop system. Moreover, the controller design explicitly accounts for partial state measurements by employing an observer to estimate the unmeasured states. The proposed conditions reduce conservatism for several reasons. A decoupling technique is employed that alleviates the limitations of the singular value decomposition approach, as the Lyapunov matrix is not restricted to a specific structure. In addition, the decision variables, essentially the controller gains, are not constant but are allowed to be polynomial functions, computed using the SOSTOOLS framework rather than the standard LMI toolbox. Furthermore, recently proposed relaxed conditions for parameterized Linear Matrix Inequalities (LMIs) in double-sum form are extended to parameterized Sum-of-Squares (SOS) constraints. The effectiveness of the proposed results is shown through two numerical examples.

## Introduction

In recent times, fractional-order models have received widespread attention owing to their outstanding in modeling systems with delays and memory effects^1–4^. Among the various fractional derivatives, the Caputo derivative was introduced by M. Caputo^5^, the Atangana–Baleanu derivative was proposed by A. Atangana and D. Baleanu^6^ and the conformable derivative was developed by Khalil et al.^7^. Each of these definitions offers special benefits depending on the application context. For instance, the conformable derivative has demonstrated improved accuracy in modeling certain practical systems such as electrical circuits^8^ and projectile motion^9^. Building on these modeling capabilities, the conformable derivative has been extensively applied to various control problems for different classes of systems. In^10^, researchers tackle the challenge of designing $H_\infty /H_-$ fault detection observers for conformable systems with locally Lipschitz nonlinearities. Reference^11^ presents a rigorous investigation into the exponential practical stability problem for linear conformable system with time delay and additive bounded uncertainty. Reference^9^ investigates the uniform asymptotic stability of time-delayed conformable neural networks with uncertainties, guaranteeing the combined $H_\infty /$passivity performance^12^. Underpinning these advancements is a thorough investigation of the conformable derivative’s mathematical properties, as conducted by scholars in^13–15^.

Meanwhile, modeling via the Takagi-Sugeno Fuzzy (TSF) framework exhibits exceptional efficacy in approximating Nonlinear Dynamical Systems (NDSs), as it represents nonlinear dynamics through local linear models blended via fuzzy rules^16^. This architecture enables accurate modeling by decomposing nonlinear behaviors into simpler subsystems. Extensively utilized in control problems for integer-order systems, the TSF model facilitates robust controller design and stability analysis^17–20^, often leveraging the Linear Matrix Inequality (LMI) toolbox^21^ and Lyapunov theory^22^ to mathematically formulate and analytically solve control problems efficiently. For example^23^, presents a two-step interval estimation method for discrete-time TSF nonlinear systems with parameter uncertainties, employing a novel observer design without redundant parameters. The networked control problem for various classes of TSF model is studied using an event-triggered scheme in^24^ and a novel self-triggered mechanism in^25^. Furthermore^26^, addresses fault estimation, while^27^ focuses on fault detection in TSF models. Recently, this powerful framework has also been extended to fractional-order systems, where the TSF model, supported by LMI-based techniques and Lyapunov-based stability analysis, addresses complex analysis and control challenges. By adapting these tools to the dynamics of fractional-order systems, researchers have advanced precise modeling and effective control strategies in fields such as engineering and applied mathematics. For example, Reference^28^ develops a resilient fuzzy control strategy for non-integer-order hydroelectric turbine represented by TSF models, considering the effects of random disturbances. In^29^, the authors develop an observer-driven control scheme for TSF models governed by the Hadamard non-integer-order derivative, ensuring the $H_\infty$ performance. Reference^30^ investigates fault estimation for TSF fractional-order models with faults and unknown inputs using a novel fuzzy unknown input observer.

Although the LMI tool has been a cornerstone for the analysis and control of integer-order and fractional-order NDSs modeled using the TSF framework, its main limitation lies in the conservatism. Addressing this fundamental constraint, the SOSTOOLS^31^ has evolved as a significant enhancement beyond standard LMI toolbox. This approach was initially employed in 2009^32^ for the modelling and control of NDSs described by Polynomial Fuzzy Models (PFMs), which allow greater modeling flexibility and often require fewer rules compared to TSF models. Since 2009, considerable advancements have been proposed to address various classes of integer-order PFMs, enhancing their applicability in analysis and control design^33,35^. For instance^36^, designs controllers for both dynamic output feedback and state feedback for a nonlinear marine system modeled with a PF framework. In more recent developments, PFMs have been extended to address various control problems of fractional-order NDSs using the SOS approach. Limited research has been conducted to address analysis and control challenges in fractional-order PFMs, highlighting a gap in comprehensive studies and methodologies for these models. For example in^37^, the authors explore observer-based control for PFM with Caputo derivative. Nevertheless, we observe that no rigorous SOS-based studies have been reported for Conformable PFMs with time delay. Inspired by the above observation, we seek to generalize the approach in^38^ to PFMs and apply the matrix decoupling technique to derive strict SOS conditions. Furthermore, the controller ensures strictly $(\mathcal {U},\mathcal {V},\mathcal {W})$-$\sigma$-dissipativity, a more general property that encompasses the $H_\infty$ performance, rather than directly guaranteeing the $H_\infty$ performance. This study makes three main contributions, which are outlined below.

(i) We consider a more general modeling framework for CNDSs with time delay. In this work, we employ a PF model rather than the TSF model in^38^.
(ii) Although^39^ uses PF modeling for CNDSs with time delay, it does not consider system external disturbances. In contrast, our work accounts for external disturbances and designs an observer-based controller that ensures both exponential stability and dissipativity performance. Moreover, unlike the singular value decomposition technique commonly used in the literature and particularly in^39^ as a decoupling method, we employ a method that avoids restricting the Lyapunov matrix to a specific form.
(iii) By employing the new sum relaxation method proposed in^40^, sufficient conditions are formulated as SOS.

The paper is structured as follows: Section "Preliminary concepts and problem description" presents preliminaries, covering the conformable derivative and the SOS approach. Moreover, it formulates the problem, focusing on the PFM with conformable derivative and time delay, alongside strictly $(\mathcal {U},\mathcal {V},\mathcal {W})$-$\sigma$-dissipativity performance. Section “Results” outlines the primary findings, deriving conditions using Lyapunov theory and providing an algorithm outlining the design steps. Section “Examples” offers two illustrative examples, and Section “Conclusion” concludes with perspectives.

### Notations 1

Let $\star$ denotes the symmetric transpose term; $\oslash$ denotes a block matrix of suitable size with no effect on the development; $\Vert .\Vert$ is the standard Euclidean vector norm; 0 represents either a zero matrix with suitable dimensions or the scalar zero, as dictated by the context; *diag*(.) denotes a block-diagonal matrix.

Given $n\in \mathbb {N}$ and $d\in \mathbb {R}_{>0}$, we define:

$I_n$ as the $n\times n$ identity matrix; $\mathbb {I}_n\triangleq \{1, \ldots ,\;n\}$;

$C\big ([-\textrm{d}, 0], \mathbb {R}^{n} \big )$ as the set of continuous functions mapping from $[-d\;0]$ to $\mathbb {R}^n$;

$$\digamma ^{n-1}\triangleq \Big \{ \eta = [\eta _1, \dots , \eta _n] \in \mathbb {R}^n \ \Big |\ \eta _i \ge 0,\; \forall i\in \mathbb {I}_n,\;\ \displaystyle \sum _{i=1}^{n} \eta _i = 1 \Big \}.$$

Given $\xi \in \mathbb {R}^{n}$, $(p,q)\in \mathbb {N}\times \mathbb {N}$, we define:

$\mathbb {R}[\xi ]$, $\mathbb {R}_{\ge 0}[\xi ]$, $\mathbb {R}^p[\xi ]$, $\mathbb {R}^{p\times q}[\xi ]$ and $\textstyle \sum \nolimits _\xi$ as the sets of polynomials, nonnegative polynomials, polynomial vectors of size *p*, polynomial matrices of size $p\times q$ and SOS in $\xi$, respectively;

$$\mathbb {S}^{p\times p}\triangleq \{\mathcal {K}\in \mathbb {R}^{p\times p}\;|\; \mathcal {K}=\mathcal {K}^T\}$$, $$\mathbb {S}^{p\times p}[\xi ]\triangleq \{\mathcal {K}(\xi )\in \mathbb {R}^{p\times p}[\xi ]\;|\; \mathcal {K}(\xi )=\mathcal {K}(\xi )^T\}$$.

## Preliminary concepts and problem description

### Preliminary concepts

#### Definition 2.1

^15^ Let $\xi : [\tau _0,\infty )\longrightarrow \mathbb {R}$ be a sufficiently smooth function. The conformable derivative of $\xi$ is given by its definition as follows:

2.1 $$\begin{aligned} T^\beta _{\tau _0}\xi (\tau )=\lim _{\varpi \rightarrow 0}\frac{\xi \left( \tau +\varpi (\tau -\tau _0)^{1-\beta }\right) -\xi (\tau )}{\varpi },\; \forall \tau>\tau _0,\; 0<\beta \le 1. \end{aligned}$$

Given that $\forall \tau \in (\tau _0,\textrm{b})$ where $\textrm{b}>\tau _0$, $T^\beta _{\tau _0}\xi (\tau )$ and $\lim _{\tau \longrightarrow \tau _0^+}T^\beta _{\tau _0}\xi (\tau )$ exist, then, by definition

$$\begin{aligned} T^\beta _{\tau _0}\xi (\tau _0)=\lim _{\tau \longrightarrow \tau _0^+}T^\beta _{\tau _0}\xi (\tau ). \end{aligned}$$

#### Definition 2.2

^15^ The conformable integral of order $\beta \in (0,1)$ for function $\xi$, taken from point $\tau _0$, is defined as

$$\begin{aligned} I^\beta _{\tau _0}\xi (\tau )=\int ^{\tau }_{\tau _0} (\tau -\tau _0)^{\beta -1} \xi (s) ds. \end{aligned}$$

#### Remark 1

When $\tau _0=0$, the definitions of the conformable fractional-order integral and derivative simplify to those provided in^7^.

Throughout the remainder of the paper, we denote: $T^{\beta }:=T^\beta _{0}$ and $I^{\beta }:=I^\beta _{0}$.

We summarize the following properties of the conformable derivative, which are essential for the design of the controller.

#### Lemma 1

^15^ Consider two functions $\xi _1(\tau )$ and $\xi _2(\tau )$, which are $\beta$-differentiable at $\tau>0$. Hence

i. $$T^{\beta } (\epsilon _1 \xi _1(\tau )+ \epsilon _2\xi _2(\tau ))=\epsilon _1 T^{\beta } (\xi _1(\tau ))+ \epsilon _2 T^{\beta } (\xi _2(\tau ))$$, $$\forall \epsilon _1,\; \epsilon _2 \in \mathbb {R}$$,
ii. $$T^{\beta } (\xi _1(\tau )\xi _2(\tau ))=\xi _1(\tau )T^{\beta } (\xi _2(\tau ))+\xi _2(\tau )T^{\beta } (\xi _1(\tau ))$$,
iii. Furthermore, assume that $\xi _1(\tau )$ is differentiable, thus $T^{\beta }\xi _1(\tau ) = \tau ^{1-\beta } \frac{d\xi _1(\tau )}{d\tau }$.

From i. and ii., we deduce that $\forall \xi (\tau )\in \mathbb {R}^{n}$ consists of $\beta$-differentiable functions:

2.2 $$\begin{aligned} T^{\beta }(\xi (\tau )^T \mathcal {K} \xi (\tau ))=(T^{\beta }\xi (\tau ))^T\mathcal {K} \xi (\tau )+\xi (\tau )\mathcal {K}(T^{\beta }\xi (\tau )),\; \forall \mathcal {K}\in \mathbb {R}^{n\times n}. \end{aligned}$$

#### Definition 2.3

^32^ Consider $P(\xi )\in \mathbb {R}[\xi ]$, where $\xi \in \mathbb {R}^n$. $P(\xi )\in \sum _\xi$, if there are $P_1(\xi ), \ldots ,P_m(\xi )\in \mathbb {R}[\xi ]$ so that

2.3 $$\begin{aligned} P(\xi )= \sum \limits _{i = 1}^m {P_i^2(\xi )}. \end{aligned}$$

It is evident that $P(\xi )\in \sum _\xi$ implies $P(\xi )\ge 0.$

#### Lemma 2

^32^ Consider $$\xi \in \mathbb {R}^n$$, $$\textrm{v}\in \mathbb {R}^p$$ and $$P(\xi )\in \mathbb {S}^{p\times p}[\xi ]$$.

If

2.4 $$\begin{aligned} \textrm{v}^T P(\xi )\textrm{v} \in \textstyle \sum \nolimits _{\textrm{v},\xi },\end{aligned}$$

then

2.5 $$\begin{aligned} P(\xi )\ge 0. \end{aligned}$$

#### Lemma 3

^17^ Consider $\xi \in \mathbb {R}^n$, for any $\textrm{q}_1(\xi ),\;\textrm{q}_2(\xi )\in \mathbb {R}^p[\xi ]$, we have

2.6 $$\begin{aligned} 2 \textrm{q}_1^T(\xi ) \textrm{q}_2(\xi ) \le \textrm{q}_1^T(\xi )\textrm{q}_1(\xi )+\textrm{q}_2^T(\xi )\textrm{q}_2(\xi ). \end{aligned}$$

### Problem description

Consider the CNDS with time delay $\textrm{d}$ and initial condition $\varphi \in C\big ([-\textrm{d}, 0], \mathbb {R}^{n_\xi } \big )$, described by

2.7 $$\begin{aligned} \left\{ \begin{array}{l} T^{\beta } \xi (\tau ) =N_1\big (\xi (\tau ),\xi (\tau -\textrm{d}),\textrm{y}(\tau ),\textrm{p}(\tau )\big ),\ \tau> 0,\\ \textrm{c}(\tau )=N_2\big (\xi (\tau ),\textrm{y}(\tau )\big ),\\ \delta (\tau )=N_3\big (\xi (\tau )\big ),\\ \xi (\tau )=\varphi (\tau ),\ \tau \in [-\textrm{d}, 0], \end{array} \right. \end{aligned}$$

where $N_k$ $(k\in \mathbb {I}_3)$ are nonlinear functions; $\xi (\tau )\in \mathbb {R}^{n_\xi }$, $\xi (\tau -\textrm{d})\in \mathbb {R}^{n_\xi }$, $\textrm{c}(\tau )\in \mathbb {R}^{n_\textrm{c}}$, $\delta (\tau )\in \mathbb {R}^{n_\delta }$, $\textrm{y}(\tau )\in \mathbb {R}^{n_\textrm{y}}$ and $\textrm{p}(\tau )\in \mathbb {R}^{n_\textrm{p}}$ represent the state vector, the state delay vector, the controlled output vector, the measured output vector, the control input vector and the disturbance vector, respectively.

Assuming $\textrm{z}_j(\tau )$ $(j\in \mathbb {I}_v )$ as measurable premise variables and $f_{\iota j}$ $(\iota \in \mathbb {I}_r)$ as their fuzzy sets, the following class of time delay Conformable PFM (CPFM) with $v$ premise variables and *r* rules can be derived using the sector-nonlinearity approach:

Model Rule $\iota \; (\iota \in \mathbb {I}_r)$: If $\textrm{z}_{1}(\tau )$ is $\textrm{f}_{\iota 1}$ and $\cdots$ and $\textrm{z}_{v }(\tau )$ is $\textrm{f}_{\iota v }$ THEN

2.8 $$\begin{aligned} \left\{ \begin{array}{l} T^{\beta }\xi (\tau )= \Psi _\iota (\delta (\tau )) \xi (\tau )+\Delta _\iota (\delta (\tau )) \xi (\tau -\textrm{d})+\Upsilon _\iota (\delta (\tau )) \textrm{y}(\tau )+\Omega _{\iota }(\delta (\tau ))\textrm{p}(\tau ),\\ \textrm{c}(\tau )=\mathcal {C}_\iota (\xi (\tau )) \xi (\tau )+\mathcal {D}_\iota (\xi (\tau )) \textrm{y}(\tau ),\\ \delta (\tau )= \mathcal {M}_\iota \xi (\tau ),\\ \xi (\tau )=\varphi (\tau ),\;\;\; \tau \in [-\textrm{d},0]. \end{array} \right. \end{aligned}$$

where $$\{\Psi _\iota (\delta (\tau )),\; \Delta _\iota (\delta (\tau )) \}\in \mathbb {R}^{n_\xi \times n_\xi }[\delta (\tau )]$$, $$\Upsilon _\iota (\delta (\tau )) \in \mathbb {R}^{n_\xi \times n_\textrm{y}}[\delta (\tau )]$$, $$\Omega _\iota (\delta (\tau )) \in \mathbb {R}^{n_\xi \times n_\textrm{p}}[\delta (\tau )]$$, $$\mathcal {C}_\iota (\xi (\tau )) \in \mathbb {R}^{n_\textrm{c}\times n_\xi }[\xi (\tau )]$$, $$\mathcal {D}_\iota (\xi (\tau )) \in \mathbb {R}^{n_\textrm{c}\times n_\textrm{y}}[\xi (\tau )]$$ and $$\mathcal {M}_\iota \in \mathbb {R}^{n_\delta \times n_\xi }$$.

The overall model is given by:

2.9 $$\begin{aligned} \left\{ \begin{array}{l} T^{\beta }\xi (\tau )=\displaystyle \sum _{\iota =1}^{r} \eta _\iota (\textrm{z}(\tau )) \Big ( \Psi _\iota (\delta (\tau )) \xi (\tau )+\Delta _\iota (\delta (\tau )) \xi (\tau -\textrm{d})+\Upsilon _\iota (\delta (\tau )) \textrm{y}(\tau )+\Omega _{\iota }(\delta (\tau ))\textrm{p}(\tau )\Big )\\ \textrm{c}(\tau )=\displaystyle \sum _{\iota =1}^{r} \eta _\iota (\textrm{z}(\tau )) \Big (\mathcal {C}_\iota (\xi (\tau )) \xi (\tau )+\mathcal {D}_\iota (\xi (\tau )) \textrm{y}(\tau )\Big ),\\ \delta (\tau )=\displaystyle \sum _{\iota =1}^{r} \eta _\iota (\textrm{z}(\tau )) \mathcal {M}_\iota \xi (\tau ), \end{array} \right. \end{aligned}$$

where $\eta _\iota (\textrm{z}(\tau ))=\frac{\displaystyle \prod _{j =1}^v \textrm{f}_{\iota j }(\textrm{z}_j (\tau ))}{\displaystyle \sum _{\iota =1}^r \prod _{j =1}^v \textrm{f}_{\iota j }(\textrm{z}_j (\tau ))}$ in which $\textrm{z}(\tau )=[\textrm{z}_1(\tau ), \ldots , \textrm{z}_v (\tau )].$

One can see that $\eta (\textrm{z}(\tau ))\in \digamma ^{r-1}$, where $\eta (\textrm{z}(\tau ))=[\eta _1(\textrm{z}(\tau ))\; \ldots ,\;\eta _r(\textrm{z}(\tau ))].$

Consider the control law with gains $\mathcal {B}_{\iota }(\delta (\tau ))\in \mathbb {R}^{n_\xi \times n_\delta }[\delta (\tau )]$ and $\mathcal {A}_{\iota }(\delta (\tau ))\in \mathbb {R}^{n_\textrm{y}\times n_\xi }[\delta (\tau )]$, given by:

 Model Rule $\iota (\iota \in \mathbb {I}_r\}$: If $\textrm{z}_{1}(\tau )$ is $\textrm{f}_{\iota 1}$ and $\cdots$ and $\textrm{z}_{v }(\tau )$ is $\textrm{f}_{\iota v }$ THEN

2.10 $$\begin{aligned} \left\{ \begin{array}{l}T^{\beta }\widehat{\xi }(\tau )=\Psi _\iota (\delta (\tau ))\widehat{\xi }(\tau )+\Delta _\iota (\delta (\tau ))\widehat{\xi }(\tau -\textrm{d})+\Upsilon _\iota (\delta (\tau ))\textrm{y}(\tau )\\ \;\;\;\;\;\;\;\;\;\;\;\;\;\;\;+\Omega _{\iota }(\delta (\tau ))\textrm{p}(\tau )+\mathcal {B}_{\iota }(\delta (\tau ))(\delta (\tau )-\widehat{\delta }(\tau )),\\ \widehat{\delta }(\tau )= \mathcal {M}_\iota \widehat{\xi }(\tau ),\\ \textrm{y}(\tau )=\mathcal {A}_{\iota }(\delta (\tau ))\widehat{\xi }(\tau ),\\ \widehat{\xi }(\tau )=\widehat{\varphi }(\tau ),\;\;\; \tau \in [-\textrm{d},0], \end{array} \right. \end{aligned}$$

where $\widehat{\xi }(\tau )$ is the estimation of $\xi (\tau )$ and $\widehat{\varphi } \in C\big ([-\textrm{d}, 0], \mathbb {R}^{n_\xi } \big )$ is the initial condition.

Similar to (2.9), we get

2.11 $$\begin{aligned} \left\{ \begin{array}{l}T^{\beta }\widehat{\xi }(\tau )= \displaystyle \sum _{\iota =1}^r\eta _{\iota }(\textrm{z}(\tau ))[\Psi _\iota (\delta (\tau ))\widehat{\xi }(\tau )+\Delta _\iota (\delta (\tau ))\widehat{\xi }(\tau -\textrm{d})+\Upsilon _\iota (\delta (\tau ))\textrm{y}(\tau )\\ \;\;\;\;\;\;\;\;\;\;\;\;\;\;\;+\Omega _{\iota }(\delta (\tau ))\textrm{p}(\tau )+\mathcal {B}_{\iota }(\delta (\tau ))(\delta (\tau )-\widehat{\delta }(\tau ))],\\ \widehat{\delta }(\tau )= \displaystyle \sum _{\iota =1}^r\eta _\iota (\textrm{z}(\tau ))[\mathcal {M}_\iota \widehat{\xi }(\tau )],\\ \textrm{y}(\tau )=\displaystyle \sum _{\iota =1}^r\eta _\iota (\textrm{z}(\tau ))[\mathcal {A}_{\iota }(\delta (\tau ))\widehat{\xi }(\tau )],\\ \widehat{\xi }(\tau )=\widehat{\varphi }(\tau ),\;\;\; \tau \in [-\textrm{d},0]. \end{array} \right. \end{aligned}$$

#### Notations 2

For simplicity, we omit the time variable $\tau$ from all polynomial matrices and $\eta _{\iota }(\textrm{z}(\tau ))$ will be referred to as $\eta _{\iota }$.

The augmented formulation is described by

2.12 $$\begin{aligned} \left\{ \begin{array}{l}T^{\beta }\tilde{\xi }(\tau )=\displaystyle \sum _{\iota =1}^r\sum _{\jmath =1}^r\eta _\iota \eta _{\jmath }[\mathcal {X}_{\iota \jmath }(\delta )\tilde{\xi }(\tau )+\mathcal {Y}_{\iota }(\delta )\tilde{\xi }(\tau -\textrm{d})+\mathcal {Z}_{\iota }(\delta )\textrm{p}(\tau )],\\ \textrm{c}(\tau )=\displaystyle \sum _{\iota =1}^r\sum _{\jmath =1}^r\eta _\iota \eta _{\jmath }[\mathcal {H}_{\iota \jmath }(\delta ,\xi )\tilde{\xi }(\tau )],\\ \tilde{\xi }(\tau )=\tilde{\varphi }(\tau ),\;\;\; \tau \in [-\textrm{d},0]. \end{array} \right. \end{aligned}$$

$$\tilde{\xi }(\tau )= \left[ \begin{array}{cc} \xi (\tau )\\ \xi (\tau )-\widehat{\xi }(\tau ) \end{array}\right] ,\quad \tilde{\varphi }(\tau )= \left[ \begin{array}{cc} \varphi (\tau )\\ \varphi (\tau )-\widehat{\varphi }(\tau ) \end{array}\right] ,$$

$$\mathcal {X}_{\iota \jmath }(\delta )= \left[ \begin{array}{cc} \Psi _\iota (\delta )+\Upsilon _\iota (\delta )\mathcal {A}_{\jmath }(\delta )& -\Upsilon _\iota (\delta )\mathcal {A}_{\jmath }(\delta )\\ 0 & \Psi _\iota (\delta )-\mathcal {B}_{\iota }(\delta )\mathcal {M}_{\jmath } \end{array}\right] , \quad \mathcal {Y}_{\iota }(\delta )= \left[ \begin{array}{cc} \Delta _\iota (\delta )& 0\\ 0 & \Delta _\iota (\delta ) \end{array}\right] ,$$

$$\mathcal {Z}_{\iota }(\delta )= \left[ \begin{array}{c} \Omega _{\iota }(\delta )\\ 0 \end{array}\right] , \quad \mathcal {H}_{\iota \jmath }(\delta ,\xi )= \left[ \begin{array}{cc} \mathcal {C}_\iota (\xi )+\mathcal {D}_\iota (\xi )\mathcal {A}_{\jmath }(\delta )&-\mathcal {D}_\iota (\xi )\mathcal {A}_{\jmath }(\delta ) \end{array}\right] .$$

(2.12) takes the equivalent form:

2.13 $$\begin{aligned} \left\{ \begin{array}{l}T^{\beta }\tilde{\xi }(\tau )=\mathcal {X}(\tau )\tilde{\xi }(\tau )+\mathcal {Y}(\tau )\tilde{\xi }(\tau -\textrm{d})+\mathcal {Z}(\tau )\textrm{p}(\tau ),\\ \textrm{c}(\tau )=\mathcal {H}(\tau )\tilde{\xi }(\tau ), \end{array} \right. \end{aligned}$$

where $$\mathcal {X}(\tau )=\displaystyle \sum _{\iota =1}^r\sum _{\jmath =1}^r\eta _\iota \eta _{\jmath }[\mathcal {X}_{\iota \jmath }(\delta )],\; \mathcal {Y}(\tau )=\displaystyle \sum _{\iota =1}^r\eta _\iota [\mathcal {Y}_{\iota }(\delta )],\; \mathcal {Z}(\tau )=\displaystyle \sum _{\iota =1}^r\eta _\iota [\mathcal {Z}_{\iota }(\delta )]$$ and $$\mathcal {H}(\tau )=\displaystyle \sum _{\iota =1}^r\sum _{\jmath =1}^r\eta _\iota \eta _{\jmath }[\mathcal {H}_{\iota \jmath }(\delta ,\xi )]$$.

#### Definition 2.4

Exponential stability of model (2.13) holds if $\exists (c _1, c _2)\in \mathbb {R}^+$ such that:

2.14 $$\begin{aligned} \Vert \tilde{\xi }(\tau )\Vert \le c _1 \mathcal {E}_{\beta }(-c _2, \tau )\; \sup _{s\in [-\textrm{d}, 0]}\Vert \tilde{\varphi }(s)\Vert ,\ \forall \tau \ge 0, \end{aligned}$$

where $$\mathcal {E}_{\beta }(-c _2, \tau )=e^{-c _2 \frac{\tau ^{\beta }}{\beta }}$$.

#### Definition 2.5

The following modified energy supply function is introduced for the augmented CPFM (2.13):

2.15 $$\begin{aligned} E(\tau )=\int ^{\tau }_{0} s^{\beta -1}\textrm{c}(s)^{T}\mathcal {U}\textrm{c}(s)ds+2\int ^{\tau }_{0} s^{\beta -1}\textrm{c}(s)^{T}\mathcal {V}\textrm{p}(s) ds+\int ^{\tau }_{0} s^{\beta -1}\textrm{p}(s)^{T}\mathcal {W}\textrm{p}(s) ds,\; \forall \tau \ge 0, \end{aligned}$$

with $$\sigma \in \mathbb {R}_{>0}$$, $$\mathcal {U}\in \mathbb {S}^{n_\textrm{c}\times n_\textrm{c}}$$, $$\mathcal {V}\in \mathbb {R}^{n_\textrm{c}\times n_\textrm{p}}$$, and $$\mathcal {W}\in \mathbb {S}^{n_\textrm{p}\times n_\textrm{p}}$$.

We can assume, without restricting generality, that $\mathcal {U}\le 0$ and $-\mathcal {U}=\mathcal {U}_1^T\mathcal {U}_1$ for some $\mathcal {U}_1\ge 0$.

The system (2.13) is deemed to be strictly $(\mathcal {U},\mathcal {V},\mathcal {W})$-$\sigma$-dissipative when it satisfies the subsequent inequality under zero initial conditions:

2.16 $$\begin{aligned} E(\tau )\ge \sigma \int ^{\tau }_{0} s^{\beta -1}\textrm{p}(s)^{T}\textrm{p}(s)ds,\;\;\;\forall \tau \geqslant 0. \end{aligned}$$

## Results

### Notations 3

Let $\vartheta \in \mathbb {R}^\kappa$, $\mathcal {I}_{\iota \jmath }(\vartheta ) \in \mathbb {S}^{m\times m}[\vartheta ]$ and $\mathcal {H}_{\iota \jmath }(\vartheta ) \in \mathbb {R}^{m\times n}[\vartheta ]$ for all $(\iota ,\jmath )\in \mathbb {I}_r\times \mathbb {I}_r$, We introduce the following notations $\forall \iota \in \mathbb {I}_r,\;\forall \varphi _1, \ldots , \varphi _{r-1} \in \{0,1\}:$

3.1 $$\begin{aligned} & \boldsymbol{\mathcal {I}}_{\iota \iota }(\vartheta )\triangleq \mathcal {I}_{\iota \iota }(\vartheta )+\frac{1}{2} \sum _{\jmath =1,\jmath <\iota }^r\varphi _{\jmath }(\mathcal {I}_{\iota \jmath }(\vartheta )+\mathcal {I}_{\jmath \iota }(\vartheta ))+\frac{1}{2}\displaystyle \sum _{\jmath =1,\jmath>\iota }^r\varphi _{\jmath -1}(\mathcal {I}_{\iota \jmath }(\vartheta )+\mathcal {I}_{\jmath \iota }(\vartheta )), \end{aligned}$$

3.2 $$\begin{aligned} & [\mathcal {H}_{\iota }(\vartheta )]_1\triangleq \left[ \begin{array}{c}\frac{1}{\sqrt{2}}\varphi _{\jmath }\mathcal {H}_{\iota \jmath }(\vartheta )\end{array}\right] _{\jmath =1, \ldots ,r;\; \jmath <\iota }\triangleq \left[ \begin{array}{ccc} \frac{1}{\sqrt{2}}\varphi _{1}\mathcal {H}_{\iota 1}(\vartheta )&\ldots&\frac{1}{\sqrt{2}}\varphi _{\iota -1}\mathcal {H}_{\iota (\iota -1)}(\vartheta ) \end{array}\right] ,\end{aligned}$$

3.3 $$\begin{aligned} & [\mathcal {H}_{\iota }(\vartheta )]_2\triangleq \left[ \begin{array}{c}\frac{1}{\sqrt{2}}\varphi _{\jmath }\mathcal {H}_{\jmath \iota }(\vartheta )\end{array}\right] _{\jmath =1, \ldots ,r;\; \jmath <\iota }\triangleq \left[ \begin{array}{ccc} \frac{1}{\sqrt{2}}\varphi _{1}\mathcal {H}_{1\iota }(\vartheta )&\ldots&\frac{1}{\sqrt{2}}\varphi _{\iota -1}\mathcal {H}_{(\iota -1)\iota }(\vartheta ) \end{array}\right] ,\end{aligned}$$

3.4 $$\begin{aligned} & [\mathcal {H}_{\iota }(\vartheta )]_3\triangleq \left[ \begin{array}{c}\frac{1}{\sqrt{2}}\varphi _{\jmath -1}\mathcal {H}_{\iota \jmath }(\vartheta )\end{array}\right] _{\jmath =1, \ldots ,r;\; \jmath>\iota }\triangleq \left[ \begin{array}{ccc} \frac{1}{\sqrt{2}}\varphi _{\iota }\mathcal {H}_{\iota (\iota +1)}(\vartheta )&\ldots&\frac{1}{\sqrt{2}}\varphi _{r-1}\mathcal {H}_{\iota r}(\vartheta ) \end{array}\right] ,\end{aligned}$$

3.5 $$\begin{aligned} & [\mathcal {H}_{\iota }(\vartheta )]_4\triangleq \left[ \begin{array}{c}\frac{1}{\sqrt{2}}\varphi _{\jmath -1}\mathcal {H}_{\jmath \iota }(\vartheta )\end{array}\right] _{\jmath =1, \ldots ,r;\; \jmath>\iota }\triangleq \left[ \begin{array}{ccc} \frac{1}{\sqrt{2}}\varphi _{\iota }\mathcal {H}_{(\iota +1)\iota }(\vartheta )&\ldots&\frac{1}{\sqrt{2}}\varphi _{r-1}\mathcal {H}_{r\iota }(\vartheta ) \end{array}\right] . \end{aligned}$$

### Remark 2

The following implication is frequently employed in observer and controller synthesis for PF models.

If

3.6 $$\begin{aligned} \mathcal {I}_{\iota \jmath }(\vartheta ) + \mathcal {I}_{\jmath \iota }(\vartheta ) < 0,\; \forall \; 1\le \iota \le \jmath \le r,\end{aligned}$$

then

3.7 $$\begin{aligned} \sum _{\iota =1}^r \sum _{\jmath =1}^r\eta _{\iota } \eta _\jmath \mathcal {I}_{\iota \jmath }(\vartheta ) < 0,\;\forall \eta _\iota \in \digamma ^{r-1}. \end{aligned}$$

In the following, we propose relaxed conditions that guarantee (3.7). While these conditions are established in^40^ for constant matrices, they are extended in the following Lemma to the case of polynomial matrices.

### Lemma 4

Let $\vartheta \in \mathbb {R}^\kappa$, $\mathcal {I}_{\iota \jmath }(\vartheta ) \in \mathbb {S}^{m\times m}[\vartheta ]$ for all $(\iota ,\jmath )\in \mathbb {I}_r\times \mathbb {I}_r$.

If

3.8 $$\begin{aligned} \boldsymbol{\mathcal {I}}_{\iota \iota }(\vartheta )< 0, \end{aligned}$$

then, we get (3.7).

### Proof

Following a similar line of reasoning as in the proof of Theorem 1 in^40^, we obtain

3.9 $$\begin{aligned} \varsigma ^T\Big (\sum _{\iota =1}^r \sum _{\jmath =1}^r\eta _{\iota } \eta _\jmath \mathcal {I}_{\iota \jmath }(\vartheta ) \Big )\varsigma \le \sum _{\iota =1}^r \eta _{\iota }^2 \Big (\varsigma ^T\mathcal {I}_{\iota \iota }(\vartheta ) \varsigma +\frac{1}{2}\sum _{\jmath =1,\jmath \ne \iota }^r max\{\varsigma ^T(\mathcal {I}_{\iota \jmath }(\vartheta )+\mathcal {I}_{\jmath \iota }(\vartheta ))\varsigma ,\;0\} \Big ), \end{aligned}$$

$$\forall \varsigma \in \mathbb {R}^m$$ independent of $$\vartheta$$, $$\varsigma \ne 0$$.

To remove the max operator from the right-hand side, we analyze all possible cases, leading to the desired conclusion:

(3.8) $$\Rightarrow \; \displaystyle \sum _{\iota =1}^r \eta _{\iota }^2 \Big (\varsigma ^T\mathcal {I}_{\iota \iota }(\vartheta ) \varsigma +\frac{1}{2}\sum _{\jmath =1,\jmath \ne \iota }^r max\{\varsigma ^T(\mathcal {I}_{\iota \jmath }(\vartheta )+\mathcal {I}_{\jmath \iota }(\vartheta ))\varsigma ,\;0\} \Big )<0$$

$$\Rightarrow \; \varsigma ^T\Big (\displaystyle \sum _{\iota =1}^r \sum _{\jmath =1}^r\eta _{\iota } \eta _\jmath \mathcal {I}_{\iota \jmath }(\vartheta ) \Big )\varsigma <0 \Leftrightarrow$$ (3.7). $$\square$$

The following Theorem provides sufficient conditions under which the system (2.13) achieves exponential stability for $\textrm{p}(\tau )=0$ while satisfying the strictly $(\mathcal {U},\mathcal {V},\mathcal {W})$-$\sigma$-dissipativity for any nonzero $\textrm{p}(\tau )$.

### Theorem 3.1

For given $\alpha \in \mathbb {R}_{>0}$, the system (2.13) is exponentially stable and strictly $(\mathcal {U},\mathcal {V},\mathcal {W})$-$\sigma$-dissipative, if there exist $\mathcal {K}, \mathcal {L}\in \mathbb {S}^{2n_\xi \times 2n_\xi }$ such that

3.10 $$\begin{aligned} & \mathcal {K}>0,\; \mathcal {L}>0,\end{aligned}$$

3.11 $$\begin{aligned} & \left[ \begin{array}{ccccc} \boldsymbol{\tilde{\mathcal {I}}}_{\iota \iota }(\delta ,\xi )& \Gamma _{\iota }(\delta ,\xi )\\ \star & -I_{N } \end{array}\right] <0,\; \forall \iota \in \mathbb {I}_r, \end{aligned}$$

where $$N =(2r-1)n_c$$,

$$\begin{aligned} \tilde{\mathcal {I}}_{\iota \jmath }(\delta ,\xi )= & \left[ \begin{array}{ccc} \left. \begin{array}{c}\mathcal {K}\mathcal {X}_{\iota \jmath }(\delta )+\mathcal {X}^{T}_{\iota \jmath }(\delta )\mathcal {K}\\ +\mathcal {L}+2\alpha \mathcal {K} \end{array}\right. & \mathcal {K} \mathcal {Y}_{\iota }(\delta )& \mathcal {K}\mathcal {Z}_{\iota }(\delta )-\mathcal {H}_{\iota \jmath }(\delta ,\xi )^T \mathcal {V}^T\\ \star & -e^{-2\alpha \frac{\textrm{d}^{\beta }}{\beta }} \mathcal {L}& 0\\ \star & \star & -(\mathcal {W}-\sigma I_{n_p}) \end{array}\right] , \end{aligned}$$

$$\begin{aligned} \Gamma _{\iota }(\delta ,\xi )=\left[ \begin{array}{ccccc}\tilde{\mathcal {H}}_{\iota \iota }(\delta ,\xi )^T&[\tilde{\mathcal {H}}_{\iota }(\delta ,\xi )^T]_{1}&[\tilde{\mathcal {H}}_{\iota }(\delta ,\xi )^T]_{2}&[\tilde{\mathcal {H}}_{\iota }(\delta ,\xi )^T]_{3}&[\tilde{\mathcal {H}}_{\iota }(\delta ,\xi )^T]_{4} \end{array}\right] , \end{aligned}$$

in which

$$\begin{aligned} \tilde{\mathcal {H}}_{\iota \jmath }(\delta ,\xi )= & \left[ \begin{array}{ccc} \mathcal {U}_1 \mathcal {H}_{\iota \jmath }(\delta ,\xi )&0&0 \end{array}\right] . \end{aligned}$$

Furthermore, $\tilde{\xi }(\tau )$ satisfies (2.14), where

3.12 $$\begin{aligned} c_1=\sqrt{\frac{\lambda _{max}(\mathcal {K})}{\lambda _{min}(\mathcal {K})}+ \frac{\lambda _{max}(\mathcal {L})}{2\alpha \lambda _{min}(\mathcal {K})} (1-e^{-2\alpha \frac{d^\beta }{\beta }})},\; c_2=\alpha . \end{aligned}$$

### Proof

Consider the subsequent Lyapunov-Krasovskii functional candidate

3.13 $$\begin{aligned} \mathscr {V}(\tau )=\mathscr {V}_1(\tau )+\mathscr {V}_2(\tau ),\end{aligned}$$

where

$$\begin{aligned}\mathscr {V}_1(\tau )=\tilde{\xi }^T(\tau ) \mathcal {K} \tilde{\xi }(\tau ),\; \mathscr {V}_2(\tau )=\int _\tau ^{\tau +\textrm{d}} s ^{\beta -1} e^{2\alpha (\frac{s ^{\beta }}{\beta }-\frac{\tau ^{\beta }}{\beta }-\frac{\textrm{d}^{\beta }}{\beta })}\tilde{\xi }^T(s -\textrm{d}) \mathcal {L} \tilde{\xi }(s -\textrm{d}) ds . \end{aligned}$$

Using item iii. of Lemma 1 along with the Leibniz integral rule, we get:

3.14 $$\begin{aligned} T^{\beta } \mathscr {V}_2(\tau )= & \tau ^{1-\beta } \Big ( (\tau +\textrm{d})^{\beta -1} e^{2\alpha \frac{(\tau +\textrm{d})^{\beta }-\tau ^{\beta }-\textrm{d}^{\beta }}{\beta }}\; \tilde{\xi }(\tau )^T \mathcal {L} \tilde{\xi }(\tau ) \nonumber \\ & - \tau ^{\beta -1} e^{{-2\alpha \frac{\textrm{d}^{\beta }}{\beta }}} \; \tilde{\xi }^T(\tau -\textrm{d}) \mathcal {L} \tilde{\xi }(\tau -\textrm{d})-2\alpha \tau ^{\beta -1} \mathscr {V}_2(\tau )\Big ). \end{aligned}$$

Since the order of the conformable derivative satisfies $0<\beta \le 1$ as given in Definition 2.1 originally proposed in^15^, the function $x^\beta$ is subadditive for $x\ge 0$. Therefore, we have

3.15 $$\begin{aligned} (\tau +\textrm{d})^{\beta }\le \tau ^{\beta }+\textrm{d}^{\beta }, \end{aligned}$$

which implies

3.16 $$\begin{aligned} T^{\beta } \mathscr {V}_2(\tau )\le & \tilde{\xi }(\tau )^T \mathcal {L} \tilde{\xi }(\tau )-e^{{-2\alpha \frac{\textrm{d}^{\beta }}{\beta }}} \; \tilde{\xi }^T(\tau -\textrm{d}) \mathcal {L} \tilde{\xi }(\tau -\textrm{d})-2\alpha \mathscr {V}_2(\tau ). \end{aligned}$$

For $\tau>0$, we get

3.17 $$\begin{aligned} T^{\beta } \mathscr {V}(\tau )\le & 2 \tilde{\xi }^T(\tau ) \mathcal {K} \Big (\mathcal {X}(\tau ) \tilde{\xi }(\tau ) + \mathcal {Y}(\tau ) \tilde{\xi }(\tau -\textrm{d})+\mathcal {Z}(\tau ) \textrm{p}(\tau )\Big ) \nonumber \\ & + \tilde{\xi }^T(\tau ) \mathcal {L} \tilde{\xi }(\tau )- e^{-2\alpha \frac{\textrm{d}^{\beta }}{\beta }} \tilde{\xi }^T(\tau -\textrm{d}) \mathcal {L} \tilde{\xi }(\tau -\textrm{d})-2\alpha \mathscr {V}_2(\tau ). \end{aligned}$$

Let

$$\begin{aligned} J(\tau )=T^{\beta } \mathscr {V}(\tau )+ 2 \alpha \mathscr {V}(\tau )-\textrm{c}(\tau )^{T}\mathcal {U} \textrm{c}(\tau )-2\textrm{p}(\tau )^{T}\mathcal {V} \textrm{c}(\tau )-\textrm{p}(\tau )^{T}(\mathcal {W}-\sigma I_{n_p})\textrm{p}(\tau ), \end{aligned}$$

and

$$\begin{aligned} \zeta ^{T}(\tau )=[\tilde{\xi }^{T}(\tau ), \\ \tilde{\xi }^{T}(\tau -\textrm{d}), \textrm{p}^{T}(\tau )]^{T}. \end{aligned}$$

Then, we obtain

3.18 $$\begin{aligned} \begin{array}{rl}&J(\tau )\le \zeta ^{T}(\tau )[\tilde{\mathcal {I}}(\tau )+\tilde{\mathcal {H}}^{T}(\tau )\tilde{\mathcal {H}}(\tau )]\zeta (\tau ), \end{array}\end{aligned}$$

where $$\tilde{\mathcal {I}}(\tau )=\displaystyle \sum _{\iota =1}^r\sum _{\jmath =1}^r\eta _\iota \eta _{\jmath }\tilde{\mathcal {I}}_{\iota \jmath }(\delta ,\xi )$$ and $$\tilde{\mathcal {H}}(\tau )=\displaystyle \sum _{\iota =1}^r\eta _\iota \tilde{\mathcal {H}}_{\iota }(\delta ,\xi )$$.

It follows that

3.19 $$\begin{aligned} & \zeta ^T(\tau )\tilde{\mathcal {H}}^{T}(\tau )\tilde{\mathcal {H}}(\tau )\zeta (\tau )=\zeta ^T(\tau )\big (\displaystyle \sum _{\iota =1}^r\sum _{\jmath =1}^r\eta _{\iota }\eta _{\jmath } \tilde{\mathcal {H}}_{\iota \jmath }(\delta ,\xi )^T\big )\big (\sum _{\iota =1}^r\sum _{\jmath =1}^r\eta _{\iota }\eta _{\jmath } \tilde{\mathcal {H}}_{\iota \jmath }(\delta ,\xi )\big )\zeta (\tau )\end{aligned}$$

3.20 $$\begin{aligned} & =\displaystyle \sum _{\iota =1}^r\sum _{\jmath =1}^r \sum _{i=1}^r\sum _{j=1}^r \eta _{\iota }\eta _{\jmath }\eta _{i}\eta _j [\zeta ^T(\tau ) \tilde{\mathcal {H}}_{\iota \jmath }^T(\xi ) \tilde{\mathcal {H}}_{ij}(\xi ) \zeta (\tau )]. \end{aligned}$$

Applying Lemma 3 yields for any $\iota ,\;\jmath ,\;i,\;j\;\in \mathbb {I}_r$:

3.21 $$\begin{aligned} 2\zeta ^T(\tau )\tilde{\mathcal {H}}_{\iota \jmath }(\delta ,\xi )^T\tilde{\mathcal {H}}_{ij}(\xi )\zeta (\tau )\le \zeta ^T(\tau )\tilde{\mathcal {H}}_{\iota \jmath }(\delta ,\xi )^T\tilde{\mathcal {H}}_{\iota \jmath }(\delta ,\xi )\zeta (\tau )+ \zeta ^T(\tau )\tilde{\mathcal {H}}^T_{ij}(\xi )\tilde{\mathcal {H}}_{ij}(\xi )\zeta (\tau ).\end{aligned}$$

Given that $\displaystyle \sum _{\iota =1}^{r} \eta _\iota = 1$, we get:

3.22 $$\begin{aligned} \displaystyle \sum _{\iota =1}^r\sum _{\jmath =1}^r \sum _{i=1}^r\sum _{j=1}^r \eta _{\iota }\eta _{\jmath }\eta _{i}\eta _j \tilde{\mathcal {H}}_{\iota \jmath }(\delta ,\xi )^T\tilde{\mathcal {H}}_{\iota \jmath }(\delta ,\xi )=\displaystyle \sum _{\iota =1}^r\sum _{\jmath =1}^r \eta _{\iota }\eta _{\jmath } \tilde{\mathcal {H}}_{\iota \jmath }(\delta ,\xi )^T\tilde{\mathcal {H}}_{\iota \jmath }(\delta ,\xi ). \end{aligned}$$

Since $\eta _\iota \ge 0,\; \forall \iota \in \mathbb {I}_r$, it follows from (3.21) and (3.22) that

3.23 $$\begin{aligned} \zeta ^T(\tau )\Big \{ \displaystyle \sum _{\iota =1}^r\sum _{\jmath =1}^r \sum _{i=1}^r\sum _{j=1}^r \eta _{\iota }\eta _{\jmath }\eta _{i}\eta _j \tilde{\mathcal {H}}_{\iota \jmath }^T(\xi ) \tilde{\mathcal {H}}_{ij}(\xi ) \Big \}\zeta (\tau ) \le \zeta ^T(\tau )\Big \{\displaystyle \sum _{\iota =1}^r\sum _{\jmath =1}^r\eta _\iota \eta _{\jmath } \tilde{\mathcal {H}}_{\iota \jmath }^T(\xi ) \tilde{\mathcal {H}}_{\iota \jmath }(\delta ,\xi )\Big \}\zeta (\tau ),\end{aligned}$$

which leads to

3.24 $$\begin{aligned} \begin{array}{rl}&J(\tau )\le \zeta ^T(\tau )\Big \{\displaystyle \sum _{\iota =1}^r\sum _{\jmath =1}^r \eta _\iota \eta _{\jmath }\bar{\mathcal {I}}_{\iota \jmath }(\delta ,\xi )\Big \}\zeta (\tau ), \end{array}\end{aligned}$$

where $$\bar{\mathcal {I}}_{\iota \jmath }(\delta ,\xi )=\tilde{\mathcal {I}}_{\iota \jmath }(\delta ,\xi )+\tilde{\mathcal {H}}_{\iota \jmath }(\delta ,\xi )^T\tilde{\mathcal {H}}_{\iota \jmath }(\delta ,\xi )$$.

On the other hand, employing the Schur complement, (3.11) is equivalent to

3.25 $$\begin{aligned} \boldsymbol{\bar{\mathcal {I}}}_{\iota \jmath }(\delta ,\xi )<0.\end{aligned}$$

In view of Lemma 4, (3.25) implies that

3.26 $$\begin{aligned} \displaystyle \sum _{\iota =1}^r\sum _{\jmath =1}^r \eta _\iota \eta _{\jmath }\bar{\mathcal {I}}_{\iota \jmath }(\delta ,\xi )<0.\end{aligned}$$

Thus, if (3.11) holds, we obtain

3.27 $$\begin{aligned} T^{\beta } \mathscr {V}(\tau )-\textrm{c}(\tau )^{T}\mathcal {U} \textrm{c}(\tau )-2\textrm{p}(\tau )^{T}\mathcal {V} \textrm{c}(\tau )-\textrm{p}(\tau )^{T}(\mathcal {W}-\sigma I_{n_p})\textrm{p}(\tau )<-2 \alpha \mathscr {V}(\tau ). \end{aligned}$$

***Case 1:*** $\textrm{p}(\tau )=0$: Since $\mathcal {U}\le 0$, we deduce that

3.28 $$\begin{aligned} T^{\beta }\mathscr {V}(\tau )< -2 \alpha \mathscr {V}(\tau ),\end{aligned}$$

then

3.29 $$\begin{aligned} e^{{2\alpha \frac{\tau ^{\beta }}{\beta }}} (T^\beta \mathscr {V}(\tau )+2\alpha \mathscr {V}(\tau ))<0. \end{aligned}$$

Using items ii. and iii. of Lemma 1, we obtain

3.30 $$\begin{aligned} T^\beta (e^{{2\alpha \frac{\tau ^{\beta }}{\beta }}} \mathscr {V}(\tau ))=e^{{2\alpha \frac{\tau ^{\beta }}{\beta }}} (T^\beta \mathscr {V}(\tau )+2\alpha \mathscr {V}(\tau )), \end{aligned}$$

consequently, (3.29) implies that

3.31 $$\begin{aligned} T^\beta (e^{{2\alpha \frac{\tau ^{\beta }}{\beta }}} \mathscr {V}(\tau ))<0. \end{aligned}$$

By applying the conformable integral from 0 to $\tau$, we obtain

3.32 $$\begin{aligned} \mathscr {V}(\tau )<e^{{-2\alpha \frac{\tau ^{\beta }}{\beta }}}\mathscr {V}(0). \end{aligned}$$

By (3.13), we have

3.33 $$\begin{aligned} \mathscr {V}(0)= & \tilde{\xi }^T(0) \mathcal {K} \tilde{\xi }(0)+\int _0^{\textrm{d}} s ^{\beta -1} e^{2\alpha (\frac{s ^{\beta }}{\beta }-\frac{\textrm{d}^{\beta }}{\beta })}\tilde{\xi }^T(s -\textrm{d}) \mathcal {L} \tilde{\xi }(s -\textrm{d}) ds \nonumber \\\le & \lambda _{max}(\mathcal {K})\Vert \tilde{\xi }(0)\Vert ^2 +\lambda _{max}(\mathcal {L}) e^{-2\alpha \frac{d^\beta }{\beta }}\times \int _0^{\mathrm {\textrm{d}}} s ^{\beta -1} e^{\frac{2\alpha s^{\beta } }{\beta }}ds\times \sup _{s\in [-\textrm{d}, 0]}\Vert \tilde{\varphi }(s)\Vert ^2, \end{aligned}$$

as a result

3.34 $$\begin{aligned} \mathscr {V}(0)\le \big ( \lambda _{max}(\mathcal {K})+ \frac{\lambda _{max}(\mathcal {L})}{2\alpha } (1-e^{-2\alpha \frac{d^\beta }{\beta }})\big ) \sup _{s\in [-\textrm{d}, 0]}\Vert \tilde{\varphi }(s)\Vert ^2. \end{aligned}$$

We thus obtain (2.14), where $c_1$ and $c_2$ are given in (3.12).

***Case 2:*** $\textrm{p}(\tau )\ne 0$: We deduce that

3.35 $$\begin{aligned} T^{\beta } \mathscr {V}(\tau )-\textrm{c}(\tau )^{T}\mathcal {U} \textrm{c}(\tau )-2\textrm{p}(\tau )^{T}\mathcal {V} \textrm{c}(\tau )-\textrm{p}(\tau )^{T}(\mathcal {W}-\sigma I_{n_p})\textrm{p}(\tau )\le 0. \end{aligned}$$

Applying the conformable integral from 0 to $\tau$ with the initial condition $\tilde{\xi }(0)=0$ yields:

3.36 $$\begin{aligned} \mathscr {V}(\tau )+\sigma \int ^{\tau }_{0} s^{\beta -1}\textrm{p}(s)^{T}\textrm{p}(s)ds\le E(\tau ), \end{aligned}$$

where $E(\tau )$ is given in (2.15).

Since $\mathscr {V}(\tau )\ge 0$, we get (2.16). Hence, system (2.13) is strictly $(\mathcal {U},\mathcal {V},\mathcal {W})$-$\sigma$-dissipative. $\square$

### Theorem 3.2

The requirements in Theorem 3.1 are equivalent to:

One can find matrices $\mathcal {G}_{h},\tilde{\mathcal {L}}_{h}\in \mathbb {S}^{n_\xi \times n_\xi }$, $\tilde{\mathcal {A}}_{\jmath }(\delta )\in \mathbb {R}^{n_\textrm{y}\times n_\xi }[\delta ]$ and $\tilde{\mathcal {B}}_{\jmath }(\delta )\in \mathbb {R}^{n_\xi \times n_\delta }[\delta ]$ for $h\in \mathbb {I}_2,\;\iota \in \mathbb {I}_r$ in a manner that these inequalities are satisfied:

3.37 $$\begin{aligned} & \mathcal {G}_h>0,\; \tilde{\mathcal {L}}_h>0,\end{aligned}$$

3.38 $$\begin{aligned} & \left[ \begin{array}{cc} \boldsymbol{\mathcal {Q}}_{\iota \iota }(\delta ,\xi )& \Xi _{\iota }(\delta ,\xi )\\ \star & -I_{N } \end{array}\right] <0,\; \forall \iota \in \mathbb {I}_r,\end{aligned}$$

3.39 $$\begin{aligned} & \boldsymbol{\mathcal {R}}_{\iota \iota }(\delta )< 0,\;\forall \iota \in \mathbb {I}_r, \end{aligned}$$

where

$$\begin{aligned} \mathcal {Q}_{\iota \jmath }(\delta , \xi )=\left[ \begin{array}{ccc} \mathcal {Q}_{\iota \jmath }^{11}(\delta )& \Delta _\iota (\delta )\mathcal {G}_1& \mathcal {Q}_{\iota \jmath }^{13}(\delta , \xi )\\ \star & -e^{-2\alpha \frac{\textrm{d}^{\beta }}{\beta }}\tilde{\mathcal {L}}_1& 0\\ \star & \star & -(\mathcal {W}-\sigma I_{n_p}) \end{array}\right] , \end{aligned}$$

$$\begin{aligned} \Xi _{\iota }(\delta ,\xi )=\left[ \begin{array}{ccccc} \mathcal {S}_{\iota \iota }(\delta , \xi )^T&[\mathcal {S}_{\iota }(\delta ,\xi )^T]_{1}&[\mathcal {S}_{\iota }(\delta ,\xi )^T]_{2}&[\mathcal {S}_{\iota }(\delta ,\xi )^T]_{3}&[\mathcal {S}_{\iota }(\delta ,\xi )^T]_{4} \end{array}\right] , \end{aligned}$$

$$\begin{aligned} \mathcal {R}_{\iota \jmath }(\delta )=\left[ \begin{array}{cc} \mathcal {R}_{\iota \jmath }^{11}(\delta )& \mathcal {G}_2\Delta _\iota (\delta )\\ \star & -e^{-2\alpha \frac{\textrm{d}^{\beta }}{\beta }} \tilde{\mathcal {L}}_2 \end{array}\right] , \end{aligned}$$

in which

$$\begin{aligned} \mathcal {S}_{\iota \jmath }(\delta , \xi )= & \left[ \begin{array}{ccc}\mathcal {U}_1(\mathcal {C}_\iota (\xi )\mathcal {G}_1+\mathcal {D}_\iota (\xi )\tilde{\mathcal {A}}_{\jmath }(\delta ))&0&0 \end{array}\right] ,\\ \mathcal {Q}_{\iota \jmath }^{11}(\delta )= & \big (\Psi _\iota (\delta )\mathcal {G}_{1}+\Upsilon _\iota (\delta )\tilde{\mathcal {A}}_{\jmath }(\delta )\big )+ \big (\Psi _\iota (\delta )\mathcal {G}_{1}+\Upsilon _\iota (\delta )\tilde{\mathcal {A}}_{\jmath }(\delta )\big )^T +\tilde{\mathcal {L}}_1+2\alpha \mathcal {G}_1,\\ \mathcal {Q}_{\iota \jmath }^{13}(\delta , \xi )= & \Omega _{\iota }(\delta )-(\mathcal {C}_\iota (\xi )\mathcal {G}_1+\mathcal {D}_\iota (\xi )\tilde{\mathcal {A}}_{\jmath }(\delta ))^T\mathcal {V}^T,\\ \mathcal {R}_{\iota \jmath }^{11}(\delta )= & (\mathcal {G}_2\Psi _\iota (\delta )-\tilde{\mathcal {B}}_{\iota }(\delta )\mathcal {M}_{\jmath })+(\mathcal {G}_2\Psi _\iota (\delta )-\tilde{\mathcal {B}}_{\iota }(\delta )\mathcal {M}_{\jmath })^{T}+\tilde{\mathcal {L}}_2+2\alpha \mathcal {G}_2. \end{aligned}$$

In this case, the local polynomial gains $\mathcal {A}_{\iota }(\delta )$ and $\mathcal {B}_{\iota }(\delta )$ are given by

3.40 $$\begin{aligned} \mathcal {A}_{\iota }(\delta )=\tilde{\mathcal {A}}_{\iota }(\delta )\mathcal {G}_{1}^{-1},&\mathcal {B}_{\iota }(\delta )=\mathcal {G}_{2}^{-1}\tilde{\mathcal {B}}_{\iota }(\delta ),&\iota \in \mathbb {I}_r. \end{aligned}$$

### Proof

*Necessity*: Assume that conditions (3.11) of Theorem 3.1 are satisfied.

On one hand, we consider

3.41 $$\begin{aligned} \mathcal {K}= \left[ \begin{array}{cc} \mathcal {K}_{1}& \oslash \\ \star & \oslash \end{array}\right] , \mathcal {L}= \left[ \begin{array}{cc} \mathcal {L}_{1}& \oslash \\ \star & \oslash \end{array}\right] .\end{aligned}$$

Inserting (3.41) into (3.11) yields

3.42 $$\begin{aligned} \left[ \begin{array}{ccccc} \boldsymbol{\mathcal {P}}_{\iota \iota }(\delta ,\xi )& \tilde{\Xi }_{\iota }(\delta ,\xi )\\ \star & -I_{N } \end{array}\right] <0,\; \forall \iota \in \mathbb {I}_r, \end{aligned}$$

where

$$\begin{aligned} \mathcal {P}_{\iota \jmath }(\delta ,\xi )=\left[ \begin{array}{ccccccc} \mathcal {P}_{\iota \jmath }^{11}(\delta )& \oslash & \mathcal {K}_{1}\Delta _\iota (\delta )& \oslash & \mathcal {P}_{\iota \jmath }^{13}(\delta ,\xi )\\ \star & \oslash & \oslash & \oslash & \oslash \\ \star & \star & -e^{-2\alpha \frac{\textrm{d}^{\beta }}{\beta }}\mathcal {L}_1& \oslash & 0\\ \star & \star & \star & \oslash & \oslash \\ \star & \star & \star & \star & -(\mathcal {W}-\sigma I_{n_p}) \end{array}\right] ,\\ \tilde{\Xi }_{\iota }(\delta ,\xi )=\left[ \begin{array}{ccccc} \tilde{\mathcal {S}}_{\iota \iota }(\delta ,\xi )^T&[\tilde{\mathcal {S}}_{\iota }(\delta ,\xi )^T]_{1}&[\tilde{\mathcal {S}}_{\iota }(\delta ,\xi )^T]_{2}&[\tilde{\mathcal {S}}_{\iota }(\delta ,\xi )^T]_{3}&[\tilde{\mathcal {S}}_{\iota }(\delta ,\xi )^T]_{4} \end{array}\right] , \end{aligned}$$

in which

$$\begin{aligned} \mathcal {P}_{\iota \jmath }^{11}(\delta )= & \mathcal {K}_{1}\big (\Psi _\iota (\delta )+\Upsilon _\iota (\delta )\mathcal {A}_{\jmath }(\delta )\big )+\big (\Psi _\iota (\delta )+\Upsilon _\iota (\delta )\mathcal {A}_{\jmath }(\delta )\big )^{T}\mathcal {K}_{1}+\mathcal {L}_1+2\alpha \mathcal {K}_1, \\ \mathcal {P}_{\iota \jmath }^{13}(\delta ,\xi )= & \mathcal {K}_{1}\Omega _{\iota }(\delta )-(\mathcal {C}_\iota (\xi )+\mathcal {D}_\iota (\xi )\mathcal {A}_{\jmath }(\delta ))^T\mathcal {V}^T, \\ \tilde{\mathcal {S}}_{\iota \jmath }(\delta , \xi )= & \left[ \begin{array}{ccccc}\mathcal {U}_1(\mathcal {C}_\iota (\xi )+\mathcal {D}_\iota (\xi )\mathcal {A}_{\jmath }(\delta ))&\oslash&0&\oslash&0 \end{array}\right] .\end{aligned}$$

Consider $$\mathcal {G}_1=\mathcal {K}_1^{-1}$$ and $$\widetilde{\mathcal {L}}_1=\mathcal {K}_1^{-1}\mathcal {L}_1\mathcal {K}_1^{-1}$$.

Inequalities (3.38) are obtained by pre- and post-multiplying inequalities (3.42) with $$\left[ \begin{array}{cccccc} \mathcal {G}_{1}& 0& 0& 0& 0& 0\\ 0& 0& \mathcal {G}_{1}& 0& 0& 0\\ 0& 0& 0& 0& I_{n_p}& 0\\ 0& 0& 0& 0& 0& I_{N } \end{array}\right]$$ and its transpose.

On the other hand, by pre- and post-multiplying (3.11) by $diag(\mathcal {K}^{-1}, \mathcal {K}^{-1}, I_{n_p}, I_{N })$, we obtain

3.43 $$\begin{aligned} & \left[ \begin{array}{ccccc} \boldsymbol{\mathcal {F}}_{\iota \iota }(\delta ,\xi )& \tilde{\Gamma }_{\iota }(\delta ,\xi )\\ \star & -I_{N } \end{array}\right] <0,\; \forall \iota \in \mathbb {I}_r, \end{aligned}$$

where

$$\begin{aligned} \mathcal {F}_{\iota \jmath }(\delta ,\xi )= & \left[ \begin{array}{ccc} \mathcal {F}_{\iota \jmath }^{11}(\delta )& \mathcal {Y}_{\iota }(\delta )\mathcal {K}^{-1}& \mathcal {Z}_{\iota }(\delta )-\mathcal {K}^{-1}\mathcal {H}_{\iota \jmath }(\delta ,\xi )^T \mathcal {V}^T\\ \star & -e^{-2\alpha \frac{\textrm{d}^{\beta }}{\beta }} \mathcal {K}^{-1}\mathcal {L}\mathcal {K}^{-1}& 0\\ \star & \star & -(\mathcal {W}-\sigma I_{n_p}) \end{array}\right] ,\\ \tilde{\Gamma }_{\iota }(\delta ,\xi )= & \left[ \begin{array}{ccccc}\mathcal {K}^{-1}\tilde{\mathcal {H}}_{\iota \iota }(\delta ,\xi )^T&[\mathcal {K}^{-1}\tilde{\mathcal {H}}_{\iota }(\delta ,\xi )^T]_{1}&[\mathcal {K}^{-1}\tilde{\mathcal {H}}_{\iota }(\delta ,\xi )^T]_{2}&[\mathcal {K}^{-1}\tilde{\mathcal {H}}_{\iota }(\delta ,\xi )^T]_{3}&[\mathcal {K}^{-1}\tilde{\mathcal {H}}_{\iota }(\delta ,\xi )^T]_{4} \end{array}\right] , \end{aligned}$$

in which

$$\begin{aligned} \mathcal {F}_{\iota \jmath }^{11}(\delta )= & \mathcal {X}_{\iota \jmath }(\delta )\mathcal {K}^{-1}+\mathcal {K}^{-1}\mathcal {X}^{T}_{\iota \jmath }(\delta )+\mathcal {K}^{-1}\mathcal {L}\mathcal {K}^{-1}+2\alpha \mathcal {K}^{-1}. \end{aligned}$$

By posing $$\mathcal {K}^{-1}= \left[ \begin{array}{cc}\oslash & \oslash \\ \star & \mathcal {K}_{2} \end{array}\right]$$ and $$\mathcal {K}^{-1}\mathcal {L}\mathcal {K}^{-1}= \left[ \begin{array}{cc}\oslash & \oslash \\ \star & \mathcal {L}_2 \end{array}\right]$$, we get

3.44 $$\begin{aligned} & \left[ \begin{array}{ccccc} \boldsymbol{\tilde{\mathcal {F}}}_{\iota \iota }(\delta ,\xi )& \oslash \\ \star & \oslash \end{array}\right] <0,\; \forall \iota \in \mathbb {I}_r, \end{aligned}$$

where

$$\begin{aligned} \tilde{\mathcal {F}}_{\iota \jmath }(\delta ,\xi )=\left[ \begin{array}{ccccc} \oslash & \oslash & \oslash & \oslash & \oslash \\ \star & \tilde{\mathcal {F}}_{\iota \jmath }^{22}(\delta ) & \oslash & \Delta _\iota (\delta ) \mathcal {K}_2& \oslash \\ \star & \star & \oslash & \oslash & \oslash \\ \star & \star & \star & -e^{-2\alpha \frac{\textrm{d}^{\beta }}{\beta }} \mathcal {L}_2& \oslash \\ \star & \star & \star & \star & \oslash \end{array}\right] , \end{aligned}$$

in which $$\tilde{\mathcal {F}}_{\iota \jmath }^{22}(\delta )=(\Psi _\iota (\delta )-\mathcal {B}_{\iota }(\delta )\mathcal {M}_{\jmath })\mathcal {K}_{2}+\mathcal {K}_{2}(\Psi _\iota (\delta )-\mathcal {B}_{\iota }(\delta )\mathcal {M}_{\jmath })^{T}+\mathcal {L}_2+2\alpha \mathcal {K}_2$$.

Consider $$\mathcal {G}_2=\mathcal {K}_2^{-1}$$.

Left-multiplying (3.44) by $$\left[ \begin{array}{cccccc}0& \mathcal {G}_{2}& 0& 0& 0& 0\\ 0& 0& 0& \mathcal {G}_{2}& 0& 0 \end{array}\right]$$ and right-multiplying by its transpose, we get (3.39), where $\tilde{\mathcal {L}}_2=\mathcal {K}_2^{-1}\mathcal {L}_2\mathcal {K}_2^{-1}$.

*Sufficiently*: Let $\mathcal {A}_{\iota }(\delta )$ and $\mathcal {B}_{\iota }(\delta )$ ba as given in (3.40), and let $\mathcal {K}_{1}=\mathcal {G}_{1}^{-1}$, $\mathcal {K}_{2}=\mathcal {G}_{2}$, $\mathcal {L}_1=\mathcal {K}_1\tilde{\mathcal {L}}_1\mathcal {K}_1$ and $\mathcal {L}_2=\tilde{\mathcal {L}}_2$.

Pre- and post-multiplying (3.38) by $diag(\mathcal {K}_{1},\mathcal {K}_{1},I_{n_p},I_{N })$, we get:

3.45 $$\begin{aligned} & \left[ \begin{array}{cc} \overline{\boldsymbol{\mathcal {Q}}}_{\iota \iota }(\delta ,\xi )& \overline{\Xi }_{\iota }(\delta ,\xi )\\ \star & -I \end{array}\right] <0,\; \forall \iota \in \mathbb {I}_r, \end{aligned}$$

where

$$\begin{aligned} \overline{\mathcal {Q}}_{\iota \jmath }(\delta , \xi )= & \left[ \begin{array}{ccc} \mathcal {P}_{\iota \jmath }^{11}(\delta )& \mathcal {K}_{1}\Delta _\iota (\delta )& \mathcal {P}_{\iota \jmath }^{13}(\delta ,\xi )\\ \star & -e^{-2\alpha \frac{\textrm{d}^{\beta }}{\beta }}\mathcal {L}_1& 0\\ \star & \star & -(\mathcal {W}-\sigma I_{n_p}) \end{array}\right] ,\\ \overline{\Xi }_{\iota }(\delta ,\xi )= & \left[ \begin{array}{ccccc} \overline{\mathcal {S}}_{\iota \iota }(\delta ,\xi )^T&[\overline{\mathcal {S}}_{\iota }(\delta ,\xi )^T]_{1}&[\overline{\mathcal {S}}_{\iota }(\delta ,\xi )^T]_{2}&[\overline{\mathcal {S}}_{\iota }(\delta ,\xi )^T]_{3}&[\overline{\mathcal {S}}_{\iota }(\delta ,\xi )^T]_{4} \end{array}\right] , \end{aligned}$$

in which

$$\begin{aligned} \overline{ \mathcal {S}}_{\iota \jmath }(\delta , \xi )= & \left[ \begin{array}{ccc}\mathcal {U}_1(\mathcal {C}_\iota (\xi )+\mathcal {D}_\iota (\xi )\mathcal {A}_{\jmath }(\delta ))&0&0 \end{array}\right] . \end{aligned}$$

$\mathcal {R}_{\iota \jmath }(\delta )$ in (3.39) can be rewritten as:

$$\begin{aligned} \mathcal {R}_{\iota \jmath }(\delta )=\left[ \begin{array}{cc} \mathcal {R}_{\iota \jmath }^{11}(\delta )& \mathcal {K}_2\Delta _\iota (\delta ) \\ \star & -e^{-2\alpha \frac{\textrm{d}^{\beta }}{\beta }} \mathcal {L}_2 \end{array}\right] , \end{aligned}$$

where $$\mathcal {R}_{\iota \jmath }^{11}(\delta )= \mathcal {K}_2(\Psi _\iota (\delta )-\mathcal {B}_{\iota }(\delta )\mathcal {M}_{\jmath })+(\Psi _\iota (\delta )-\mathcal {B}_{\iota }(\delta )\mathcal {M}_{\jmath })^{T}\mathcal {K}_2+\mathcal {L}_2+2\alpha \mathcal {K}_2.$$

Let $$\Theta _{\iota \iota }(\delta )=\left[ \begin{array}{cc} \boldsymbol{\Theta }_{\iota \iota }^{11}(\delta )& 0\\ 0& 0\\ \boldsymbol{\Theta }_{\iota \iota }^{13}(\delta )& 0 \end{array}\right] ,$$ where

$$\begin{aligned}\Theta _{\iota \jmath }^{11}(\delta )=-\mathcal {K}_{1}\Upsilon _\iota (\delta )\mathcal {A}_{\jmath }(\delta ),\; \Theta _{\iota \jmath }^{13}(\delta )=\mathcal {V}\mathcal {D}_{\iota }(\delta )\mathcal {A}_{\jmath }(\delta ),\end{aligned}$$

and $$\Phi _{\iota }(\delta )=\left[ \begin{array}{cc} \Phi _{\iota }^{11}(\delta )&0 \end{array}\right] ,$$ where

$$\begin{aligned}\Phi _{\iota }^{11}(\delta )=\left[ \begin{array}{ccccc} \phi ^{11}_{\iota \iota }(\delta ,\xi )^T&[\phi ^{11}_{\iota }(\delta ,\xi )^T]_{1}&[\phi ^{11}_{\iota }(\delta ,\xi )^T]_{2}&[\phi ^{11}_{\iota }(\delta ,\xi )^T]_{3}&[\phi ^{11}_{\iota }(\delta ,\xi )^T]_{4} \end{array}\right] ^T,\end{aligned}$$

in which $$\phi _{\iota \jmath }^{11}(\delta )=-\mathcal {U}_1\mathcal {D}_{\iota }(\delta )\mathcal {A}_{\jmath }(\delta )$$.

Using the Schur complement, a scalar $\beta _{0}$ exists satisfying

3.46 $$\begin{aligned} \left[ \begin{array}{ccc} \overline{\boldsymbol{\mathcal {Q}}}_{\iota \iota }(\delta , \xi )& \overline{\Xi }_{\iota }(\delta ,\xi )& \Theta _{\iota \iota }(\delta )\\ \star & -I_{N }& \Phi _{\iota \iota }(\delta ) \\ \star & \star & \beta \boldsymbol{\mathcal {R}}_{\iota \iota }(\delta ) \end{array}\right] <0,\; \forall \beta \ge \beta _{0}. \end{aligned}$$

Pre-multiplying and post-multiplying (3.46) by $$\left[ \begin{array}{cccccc} I_{n_\xi }& 0& 0& 0& 0& 0\\ 0& 0& 0& 0& I_{n_\xi }& 0\\ 0& I_{n_\xi }& 0& 0& 0& 0\\ 0& 0& 0& 0& 0& I_{n_\xi }\\ 0& 0& I_{n_p}& 0& 0& 0\\ 0& 0& 0& I_{N }& 0& 0 \end{array}\right]$$ and its transpose, we get (3.11) for $\mathcal {K}=diag(\mathcal {K}_1,\beta \mathcal {K}_2)$ and $\mathcal {L}=diag(\mathcal {L}_1,\beta \mathcal {L}_2)$. $\square$

### Algorithm 3.3

The algorithm presented below explains the design process:

**Step 1:** Model the CNDS with time delay (2.7) as a time delay CPFM (2.9) by applying sector-nonlinearity or Taylor series approximation.

As shown in^42^, the sector-nonlinearity technique is exact for polynomial nonlinearities, with consequents closely matching the original dynamics. For smooth nonpolynomial nonlinearities (e.g., trigonometric or exponential), a Taylor-based approach allows exact PF representation within a compact domain.

**Step 2:** Let independent symbolic vectors $\delta \in \mathbb {R}^{n_\delta }$, $\xi \in \mathbb {R}^{n_\xi }$, $\textrm{v}_1\in \mathbb {R}^{n_\xi }$, $\textrm{v}_2\in \mathbb {R}^{2n_{\xi }+n_p+N }$ and $\textrm{v}_3\in \mathbb {R}^{2n_\xi }$, and let the dissipativity parameters be $\sigma \in \mathbb {R}_{>0}$, $\mathcal {U}\in \mathbb {R}^{n_\textrm{c}\times n_\textrm{c}}$, $\mathcal {V}\in \mathbb {R}^{n_\textrm{c}\times n_\textrm{p}}$, and $\mathcal {W}\in \mathbb {R}^{n_\textrm{p}\times n_\textrm{p}}$,

The following parameters are predefined for all $h\in \mathbb {I}_2$ and $\iota \in \mathbb {I}_r$ to ensure the positivity of the SOS conditions:

- $$\mu _{h}=diag(\ell _{h1},\ldots ,\ell _{hn_\xi })$$, where $$\forall s\in \mathbb {I}_{n_\xi }$$, $$\ell _{hs}\in \mathbb {R}_{>0}$$,
- $\mu _{1\iota }(\delta ,\xi )=diag(\ell _{1\iota 1}(\delta ,\xi ),\ldots ,\ell _{1\iota (2n_{\xi }+n_p)}(\delta ,\xi ),\ell _{1\iota (2n_{\xi }+n_p+1)},\ldots , \ell _{1\iota (2n_{\xi }+n_p+N )})$, where $\forall s\in \mathbb {I}_{2n_\xi +n_p}$, $\ell _{1\iota s}(\delta ,\xi )\in \mathbb {R}_{\ge 0}[\delta ,\xi ]$ such that $\ell _{1\iota s}(\delta ,\xi )>0$ for $(\delta ,\xi )\ne 0$, and $\forall s\in \{2n_\xi +n_p+1,\ldots ,2n_\xi +n_p+N \}$, $\ell _{1 \iota s}\in \mathbb {R}_{>0}$,
- $\mu _{2\iota }(\delta )=diag(\ell _{2\iota 1}(\delta ),\ldots ,\ell _{1\iota (2n_{\xi })}(\delta ))$, where $\forall s\in \mathbb {I}_{2n_\xi }$, $\ell _{2\iota s}(\delta )\in \mathbb {R}_{\ge 0}[\delta ]$ such that $\ell _{2\iota s}(\delta ,\xi )>0$ for $\delta \ne 0$,

Find $\mathcal {G}_{h},\tilde{\mathcal {L}}_{h}\in \mathbb {R}^{n_\xi \times n_\xi }$, $\tilde{\mathcal {A}}_{\iota }(\delta )\in \mathbb {R}^{n_\textrm{y}\times n_\xi }[\delta ]$ and $\tilde{\mathcal {B}}_{\iota }(\delta )\in \mathbb {R}^{n_\xi \times n_\delta }[\delta ]$ for given $\alpha \in \mathbb {R}_{>0}$ such that

3.47 $$\begin{aligned} & \textrm{v}_1^T(\mathcal {G}_h-\mu _{1})\textrm{v}_1 \in \textstyle \sum \nolimits _{\textrm{v}_1},\; \textrm{v}_1^T(\tilde{\mathcal {L}}_h-\mu _{2})\textrm{v}_1 \in \textstyle \sum \nolimits _{\textrm{v}_1},\end{aligned}$$

3.48 $$\begin{aligned} & \textrm{v}_2^T\Big (-\left[ \begin{array}{cc} \boldsymbol{\mathcal {Q}}_{\iota \iota }(\delta ,\xi )& \Xi _{\iota }(\delta ,\xi )\\ \star & -I_{N } \end{array}\right] -\mu _{1\iota }(\delta , \xi ) \Big )\textrm{v}_2\in \textstyle \sum \nolimits _{\textrm{v}_2,\delta ,\xi },\; \forall \iota \in \mathbb {I}_r,\end{aligned}$$

3.49 $$\begin{aligned} & \textrm{v}_3^T\Big (-\boldsymbol{\mathcal {R}}_{\iota \iota }(\delta )-\mu _{2\iota }(\delta )\Big )\textrm{v}_3\in \textstyle \sum \nolimits _{\textrm{v}_3,\delta },\; \forall \iota \in \mathbb {I}_r, \end{aligned}$$

where $\boldsymbol{\mathcal {Q}}_{\iota \iota }(\delta ,\xi )$, $\Xi _{\iota }(\delta ,\xi )$ and $\boldsymbol{\mathcal {R}}_{\iota \iota }(\delta )$ are defined in Theorem 3.2.

**Step 3:** Calculate the gains $\mathcal {A}_{\iota }(\delta )$ and $\mathcal {B}_{\iota }(\delta )$ based on (3.40).

**Step 4:** Determine the controller $\textrm{y}(\tau )$ based on (2.11).

**Step 5:** Implement the derived controller $\textrm{y}(\tau )$ in the original CNDS with time delay (2.7), ensuring both exponential stability and the strictly $(\mathcal {U},\mathcal {V},\mathcal {W})$-$\sigma$-dissipativity.

### Remark 3

When $\beta =1$, the conformable derivative reduces to the standard integer-order derivative, (2.14) coincides with the classical exponential stability definition where $c_1$ scales $\Vert \tilde{\xi }(\tau )\Vert$ and $c_2=\alpha$ is the exponential convergence rate.

### Remark 4

The study in^38^ tackles the problem of $H_\infty$ exponential stability for a class of nonlinear conformable systems using the TSF model and an LMI-based controller design. In contrast, our approach employs a CPFM, which offers a more compact representation of nonlinear dynamics with fewer rules, and utilizes the SOS optimization framework instead of LMIs. Moreover, rather than focusing solely on $H_\infty$ performance, we consider the more general strictly $(\mathcal {U},\mathcal {V},\mathcal {W})$-$\sigma$-dissipativity framework, which encompasses $H_\infty$ as a special case and allows for broader energy-based performance analysis.

### Remark 5

The main contributions of the proposed work, in comparison with^39^, are twofold. First, dissipativity performance is addressed in this paper, whereas it is not considered in^39^. Second, a different decoupling technique is employed. The decoupling approach in^39^, which is based on singular value decomposition, has several drawbacks. In particular, it enforces a specific structure on the Lyapunov matrix, which may increase conservatism. Moreover, the resulting conditions are linear in the polynomial variables and only imply the original bilinear polynomial conditions. In contrast, the proposed decoupling technique establishes an equivalence between the bilinear polynomial conditions and the linear polynomial conditions, thereby avoiding additional conservatism. Moreover, we extend a recently proposed relaxation technique for TSF models to the more general case of PFMs.

### Remark 6

The reduction of conservatism and the reduction of computational burden can be viewed as a trade-off. Increasing complexity is the price paid for relaxing conservative conditions, and vice versa. In our work, we seek a balanced compromise by:

(i) reducing conservatism in two ways. First, we use SOSTOOLS instead of the LMI Toolbox, which allows the gains to be polynomial matrices, thereby generalizing the case of constant gains obtained via LMIs. Second, we adopt recently developed relaxed conditions for parameterized LMIs, extending them to parameterized SOS, further reducing conservatism,
(ii) reducing computational complexity by modeling nonlinear systems with a PF model instead of a TSF model, which generally reduces the number of rules required.

### Remark 7

In general, PF models reduce the number of rules compared to TSF models, since the system matrices in the consequent part are allowed to be polynomial rather than constant. Moreover, by assuming that the system matrices depend affinely on the premise variables, a reduced-complexity polytopic representation of nonlinear systems is proposed in^41^. A key advantage of this approach is that the model complexity grows only proportionally with the number of premise variables, rather than exponentially as in conventional TSF modeling. Extending this framework to PF models, instead of TSF models, constitutes an important direction for future research.

### Discussion

This work presents several advantages in the control of CNDSs with time delay. For modeling, it uses a PF approach rather than a TSF model and accounts for external disturbances. For control, exponential stability rather than asymptotic stability is guaranteed along with dissipative performance, and an observer is also designed. In terms of design, a decoupling method is used rather than the singular value decomposition technique, which imposes restrictions on the Lyapunov matrix, and a new sum relaxation method is adopted instead of the classical relaxation. Nevertheless, the framework could be further tested on more complex problems, such as:*

(i) event-triggered control as in^24^ and self–triggered control as in^25^,
(ii) fault estimation as in^26–30^, and fault detection as in^27^,
(iii) by adopting approaches like those in^41^ to further reduce the number of rules, particularly for systems with a large number of nonlinearities.

## Examples

### Example 1

Consider a CNDS with time delay in the form of (2.7) with

$$\begin{aligned} \xi (\tau )= & \left[ \begin{array}{c} \xi _1(\tau )\\ \xi _2(\tau ) \end{array}\right] ,\; \textrm{d}=0.1,\; \beta =0.9,\; \textrm{p}(\tau )=\frac{\cos (\tau )}{(\tau +1)^2},\; \varphi (\tau )=\left[ \begin{array}{c} 3\\ 1 \end{array}\right] , \\ N_1\big (.\big )= & \left[ \begin{array}{c} -\sin (\xi _1(\tau ))-\xi _1^3(\tau )+0.9\xi _2(\tau )-0.01\xi _1^3(\tau -\textrm{d})+\textrm{y}(\tau )+0.5\textrm{p}(\tau ),\\ \nonumber 2\xi _1(\tau )-0.1\xi _2(\tau )-\xi _1(\tau )\xi _2(\tau )-0.5\xi _1^2(\tau )\xi _2(\tau )-0.01\xi _1^3(\tau -\textrm{d})+0.7\textrm{y}(\tau )+0.5\textrm{p}(\tau ) \end{array}\right] ,\\ N_2(.)= & \xi _1(\tau )+0.01\xi _1^2(\tau )+0.5\textrm{y}(\tau ),\; N_3(.)=\xi _1(\tau ). \end{aligned}$$

**Fig. 1:**
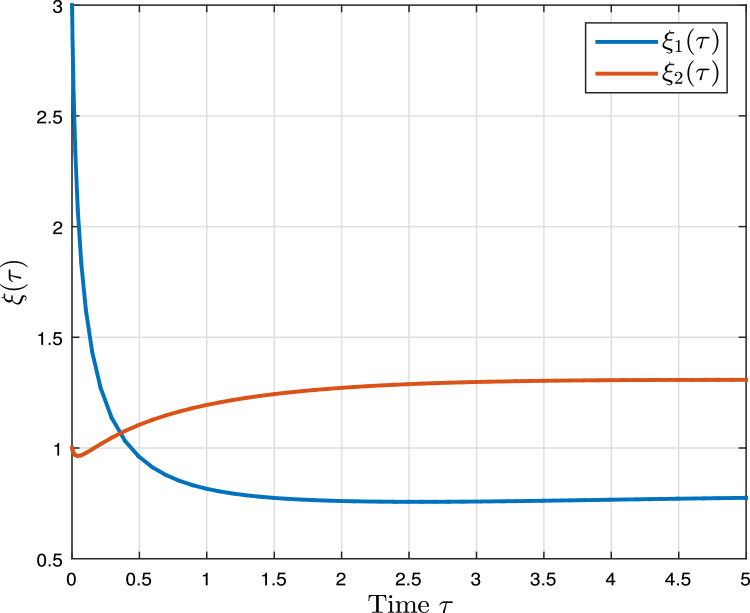
Time evolution of $\xi (\tau )$ for the CNDS in Example 1 without control $\textrm{y}(\tau )$.

Figure 1 illustrates the time evolution of $\xi (\tau )$ for the CNDS in the absence of control $\textrm{y}(\tau )$. It is evident that the open-loop CNDS does not exhibit exponential stability.

The CNDS with time delay can be represented by the following state-space form:

4.1 $$\begin{aligned} \left\{ \begin{array}{l} T^{\beta }\xi (\tau )= \Psi (\delta (\tau )) \xi (\tau )+\Delta \xi (\tau -\textrm{d})+\Upsilon \textrm{y}(\tau )+\Omega \textrm{p}(\tau ),\\ \textrm{c}(\tau )=\mathcal {C}(\delta (\tau )) \xi (\tau )+\mathcal {D} \textrm{y}(\tau ),\\ \delta (\tau )= \mathcal {M} \xi (\tau ),\\ \xi (\tau )=\varphi (\tau ),\;\;\; \tau \in [-\textrm{d},0]. \end{array} \right. \end{aligned}$$

where

$$\begin{aligned} & \Psi (\delta (\tau ))= \left[ \begin{array}{cc} -\frac{\sin (\delta (\tau ))}{\delta (\tau )}-\delta ^2(\tau )& 0.9\\ 2 & -0.1-\delta (\tau )-0.5\delta ^2(\tau ) \end{array}\right] ,\quad \Delta = \left[ \begin{array}{cc} -0.01& 0\\ 0 & -0.01 \end{array}\right] ,\\ & \nonumber \Upsilon =\left[ \begin{array}{c} 1\\ 0.7 \end{array} \right] ,\Omega =\left[ \begin{array}{c} 0.5\\ 0.5 \end{array} \right] , \quad \mathcal {C}(\delta (\tau ))= \left[ \begin{array}{cc} 1+0.01\delta (\tau )&0 \end{array} \right] ,\; \mathcal {D}=0.5,\; \mathcal {M}=\left[ \begin{array}{cc} 1&0 \end{array} \right] . \end{aligned}$$

Applying Step 1 of Algorithm 3.3 yields:

4.2 $$\begin{aligned} \left\{ \begin{array}{l} T^{\beta }\xi (\tau )= \displaystyle \sum _{\iota =1}^2\eta _{\iota }(\textrm{z}(\tau ))\Psi _\iota (\delta (\tau )) \xi (\tau )+\Delta \xi (\tau -\textrm{d})+\Upsilon \textrm{y}(\tau )+\Omega \textrm{p}(\tau ),\\ \textrm{c}(\tau )=\mathcal {C}(\delta (\tau )) \xi (\tau )+\mathcal {D} \textrm{y}(\tau ),\\ \delta (\tau )= \mathcal {M} \xi (\tau ),\\ \xi (\tau )=\varphi (\tau ),\;\;\; \tau \in [-\textrm{d},0]. \end{array} \right. \end{aligned}$$

where

$$\begin{aligned} & \Psi _1(\delta (\tau ))= \left[ \begin{array}{cc} 0.2172-\delta ^2(\tau )& 0.9\\ 2 & -0.1-\delta (\tau )-0.5\delta ^2(\tau ) \end{array}\right] ,\\ \nonumber & \Psi _2(\delta (\tau ))= \left[ \begin{array}{cc} -1-\delta ^2(\tau )& 0.9\\ 2 & -0.1-\delta (\tau )-0.5\delta ^2(\tau ) \end{array}\right] ,\\ & \nonumber \eta _{1}(\textrm{z}(\tau ))=\frac{\textrm{z}(\tau )+1}{1.2172}, \eta _{2}(\textrm{z}(\tau ))=\frac{0.2172-\textrm{z}(\tau )}{1.2172}. \end{aligned}$$

in which $$\textrm{z}(\tau )=-\frac{\sin (\delta (\tau ))}{\delta (\tau )}$$.

To represent the CNDS (4.1) using a TSF model instead of a PF model (4.2), it is necessary not only to use the premise variable $\textrm{z}(\tau )$ but also to introduce an additional premise variable $\textrm{z}_2(\tau )= \delta ^2(\tau )$. Indeed, while the TSF model uses constant matrices in the consequent part, the PF model employs polynomial matrices.

We now execute Step 2 of Algorithm 3.3 for $\sigma =1$, $\mathcal {U}=-1\; (\mathcal {U}_1=1)$, $\mathcal {V}=1$, $\mathcal {W}=3$, all slack variables are taken equal to $10^{-6}$ and $\alpha =0.9$, resulting in:

$$\mathcal {G}_{1}= \left[ \begin{array}{cc} 1.127& -0.1672\\ -0.1672& 0.2578 \end{array}\right] ,\quad \mathcal {G}_{2}=\left[ \begin{array}{cc} 2.357& -0.7696\\ -0.7696& 0.3026 \end{array} \right] ,$$

$$\tilde{\mathcal {L}}_{1}=\left[ \begin{array}{cc} 1.124& -2.2740\times 10^{-3}\\ -2.2740\times 10^{-3}& 0.4129 \end{array}\right] ,\quad \tilde{\mathcal {L}}_{2}=\left[ \begin{array}{cc} 1.098& -0.0189\\ -0.0189& 0.3112 \end{array}\right] ,$$

$$\tilde{\mathcal {A}}_1(\delta )= \left[ \begin{array}{cc} - 5.45\times 10^{-6}\delta ^2 - 2.065&3.097\times 10^{-6}\delta ^2 - 0.8108 \end{array}\right] ,$$

$$\tilde{\mathcal {A}}_2(\delta )= \left[ \begin{array}{cc} - 3.577\times 10^{-6}\delta ^2 - 1.065&2.474\times 10^{-6}\delta ^2 - 1.033 \end{array}\right] ,$$

$$\tilde{\mathcal {B}}_1(\delta )= \left[ \begin{array}{c} 2.138 - 1.556\delta ^2\\ 0.8813\delta ^2 + 1.06 \end{array}\right] , \tilde{\mathcal {B}}_2(\delta )= \left[ \begin{array}{c} - 1.603\delta ^2 - 0.5894\\ 0.913\delta ^2 + 1.931 \end{array}\right] .$$

By following Step 3 of Algorithm 3.3, we obtain

$$\mathcal {A}_1(\delta )= \left[ \begin{array}{cc} -3.3799\times 10^{-6}\delta ^2 - 2.543&9.8188\times 10^{-6}\delta ^2 - 4.7948 \end{array}\right] ,$$

$$\mathcal {A}_2(\delta )= \left[ \begin{array}{cc} -1.9371\times 10^{-6}\delta ^2 - 1.7035&8.3415\times 10^{-6}\delta ^2 - 5.1112 \end{array}\right] ,$$

$$\mathcal {B}_1(\delta )= \left[ \begin{array}{c} 1.7123\delta ^2 +12.0779\\ 7.2662\delta ^2 + 34.2168 \end{array}\right] , \mathcal {B}_2(\delta )= \left[ \begin{array}{c} 1.7952\delta ^2 +10.7929\\ 7.5819\delta ^2 + 33.8249 \end{array}\right] .$$

**Fig. 2:**
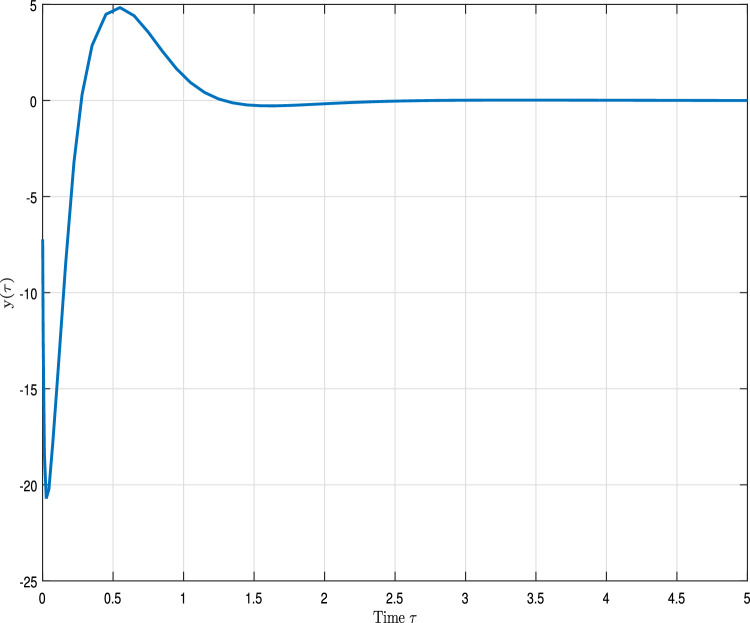
Control input $\textrm{y}(\tau )$ for the CNDS in Example 1.

Next, we execute Step 4 of Algorithm 3.3 using 2.11 with $$\widehat{\varphi }(\tau )=\left[ \begin{array}{c} 1\\ 1 \end{array}\right]$$, which yields the control input depicted in Fig. 2

**Fig. 3:**
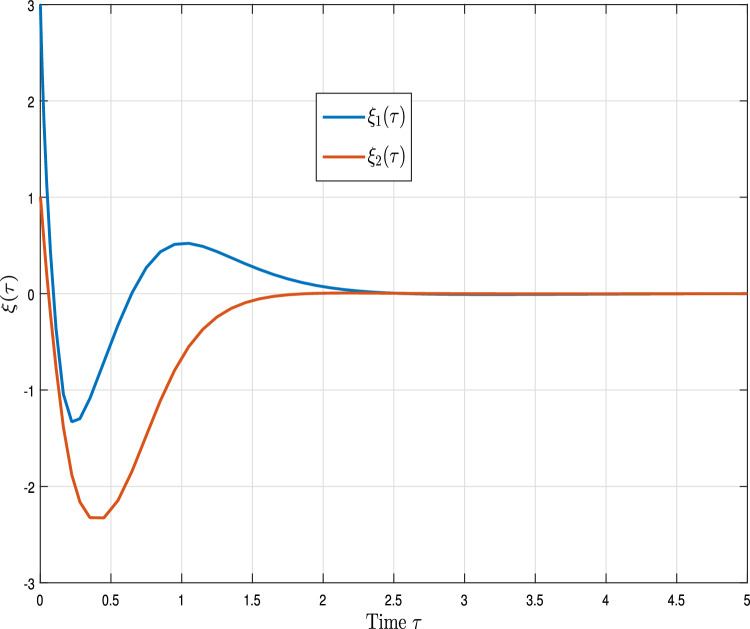
Time evolution of $\xi (\tau )$ for the CNDS with control $\textrm{y}(\tau )$ in Example 1.

Finally, step 5 of Algorithm 3.3 is carried out by applying the previously designed controller $\textrm{y}(\tau )$ to the CNDS. Figure 3 presents the closed-loop system state. Clearly, the controller $\xi (\tau )$ ensures exponential stability of the system.

**Fig. 4:**
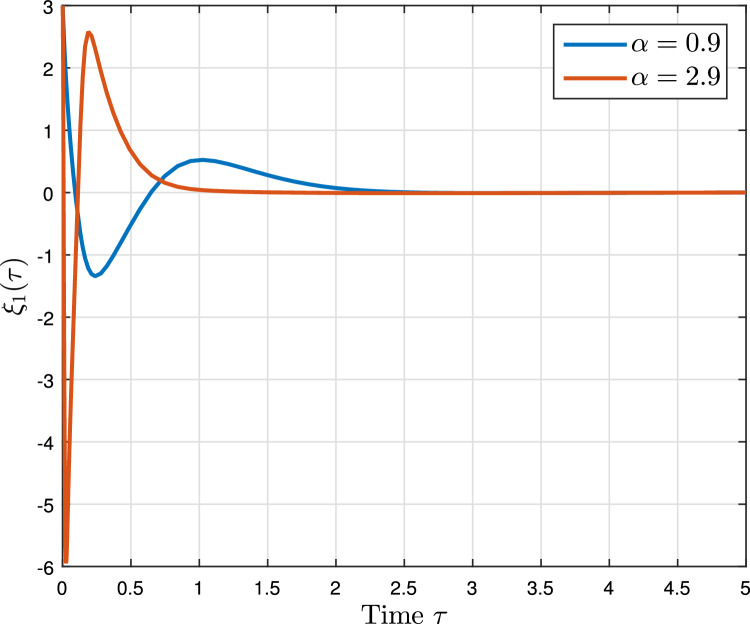
Time evolution of $\xi _1(\tau )$ for the CNDS in Example 1 with control $\textrm{y}(\tau )$ for $\alpha =0.9$ and $\alpha =2.9$.

**Fig. 5:**
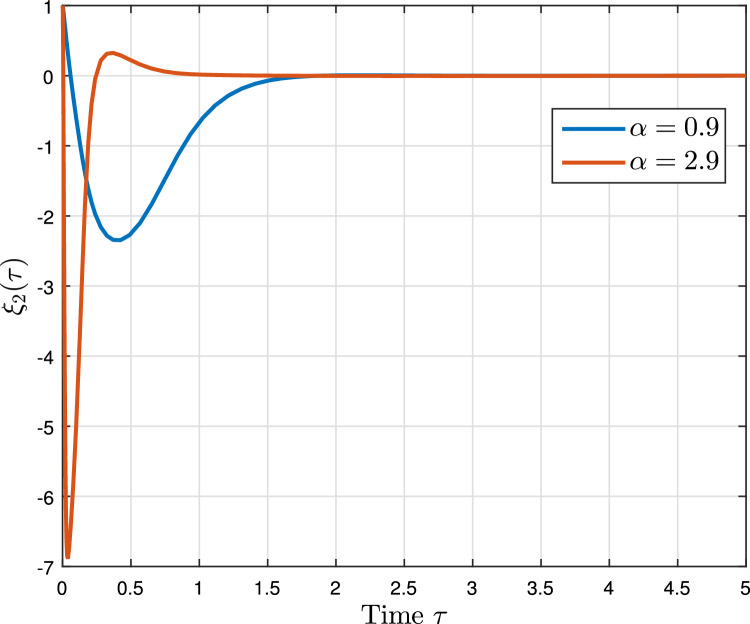
Time evolution of $\xi _2(\tau )$ for the CNDS in Example 1 with control $\textrm{y}(\tau )$ for $\alpha =0.9$ and $\alpha =2.9$.

To illustrate the effect of $c_2$, which equals $\alpha$ according to (3.12), we set $\alpha =2.9$. Figure 4 shows the time evolution of $\xi _1(\tau )$, and Fig. 5 shows that of $\xi _2$ for $\alpha =0.9$ and $\alpha =2.9$, illustrating the effect of $\alpha$ on the system’s speed of response.

### Example 2

Consider the CNDS described by (4.1), consisting of three states and four nonlinear terms, where:

$$\begin{aligned} & \Psi (\delta (\tau ))= \left[ \begin{array}{ccc} z_1(\tau )& 0.9& 0\\ 2& z_2(\tau ) & 0.5z_3(\tau )\\ 0& 0& z_4(\tau ) \end{array}\right] ,\quad \Delta = \left[ \begin{array}{ccc} -0.01& 0& 0\\ 0 & -0.01& 0\\ 0& 0& 0 \end{array}\right] ,\\ & \Upsilon =\left[ \begin{array}{c} 1\\ 0.7\\ 0 \end{array} \right] ,\Omega =\left[ \begin{array}{c} 0.5\\ 0\\ 0 \end{array} \right] , \quad \mathcal {C}= \left[ \begin{array}{ccc} 1&0&0 \end{array} \right] ,\; \mathcal {D}=0.5,\; \mathcal {M}=\left[ \begin{array}{ccc} 1& 0& 0\\ 0& 1& 0 \end{array} \right] . \end{aligned}$$

where $$z_1(\tau )=\frac{e^{-2\delta _1(\tau )}}{1+e^{-2\delta _1(\tau )}}$$, $$\;z_2(\tau )=-0.1-\delta _1(\tau )-0.5\delta _1^2(\tau )$$, $$\; z_3(\tau )=\frac{1+\sin ^2(\delta _2(\tau ))}{2}$$, $$\; z_4(\tau )=-1-\delta _2^2(\tau )$$.

Given that the output matrix is $\mathcal {M}$, the measured output vector is $$\delta (\tau )=\left[ \begin{array}{c} \delta _1(\tau )\\ \delta _2(\tau ) \end{array} \right] =\left[ \begin{array}{c} \xi _1(\tau )\\ \xi _2(\tau ) \end{array} \right]$$. Therefore, the premise variables are measurable.

Taking into account the four premise variables, the system can be modeled using a TSF framework with $2^4$ rules. However, considering only the nonpolynomial premise variables $0\le z_1(\tau )\le 1$ and $0\le z_3(\tau ) \le 1$, the system admits a PF representation with $2^2$ rules, where

$$\begin{aligned} & \Psi _1(\delta (\tau ))= \left[ \begin{array}{ccc} 1& 0.9& 0\\ 2& z_2(\tau ) & 0.5\\ 0& 0& z_4(\tau ) \end{array}\right] ,\; \Psi _2(\delta (\tau ))= \left[ \begin{array}{ccc} 1& 0.9& 0\\ 2& z_2(\tau ) & 0\\ 0& 0& z_4(\tau ) \end{array}\right] ,\\ \nonumber & \Psi _3(\delta (\tau ))= \left[ \begin{array}{ccc} 0& 0.9& 0\\ 2& z_2(\tau ) & 0.5\\ 0& 0& z_4(\tau ) \end{array}\right] ,\; \Psi _4(\delta (\tau ))= \left[ \begin{array}{ccc} 0& 0.9& 0\\ 2& z_2(\tau ) & 0\\ 0& 0& z_4(\tau ) \end{array}\right] ,\; \eta _1(\tau )=z_1(\tau )z_3(\tau ),\\ \nonumber & \eta _2(\tau )=z_1(\tau )(1-z_3(\tau )),\; \eta _3(\tau )=(1-z_1(\tau ))z_3(\tau ),\;\eta _4(\tau )=(1-z_1(\tau ))(1-z_3(\tau )). \end{aligned}$$

We take the disturbance as:

$$\textrm{p}(\tau ) = {\left\{ \begin{array}{ll} \sin \left( \frac{\pi }{2} \tau \right) , & 0 \le \tau < 2, \\ e^{-0.1 (\tau -2)} \sin \left( \frac{\pi }{2} \tau \right) , & \tau \ge 2. \end{array}\right. }$$

**Fig. 6:**
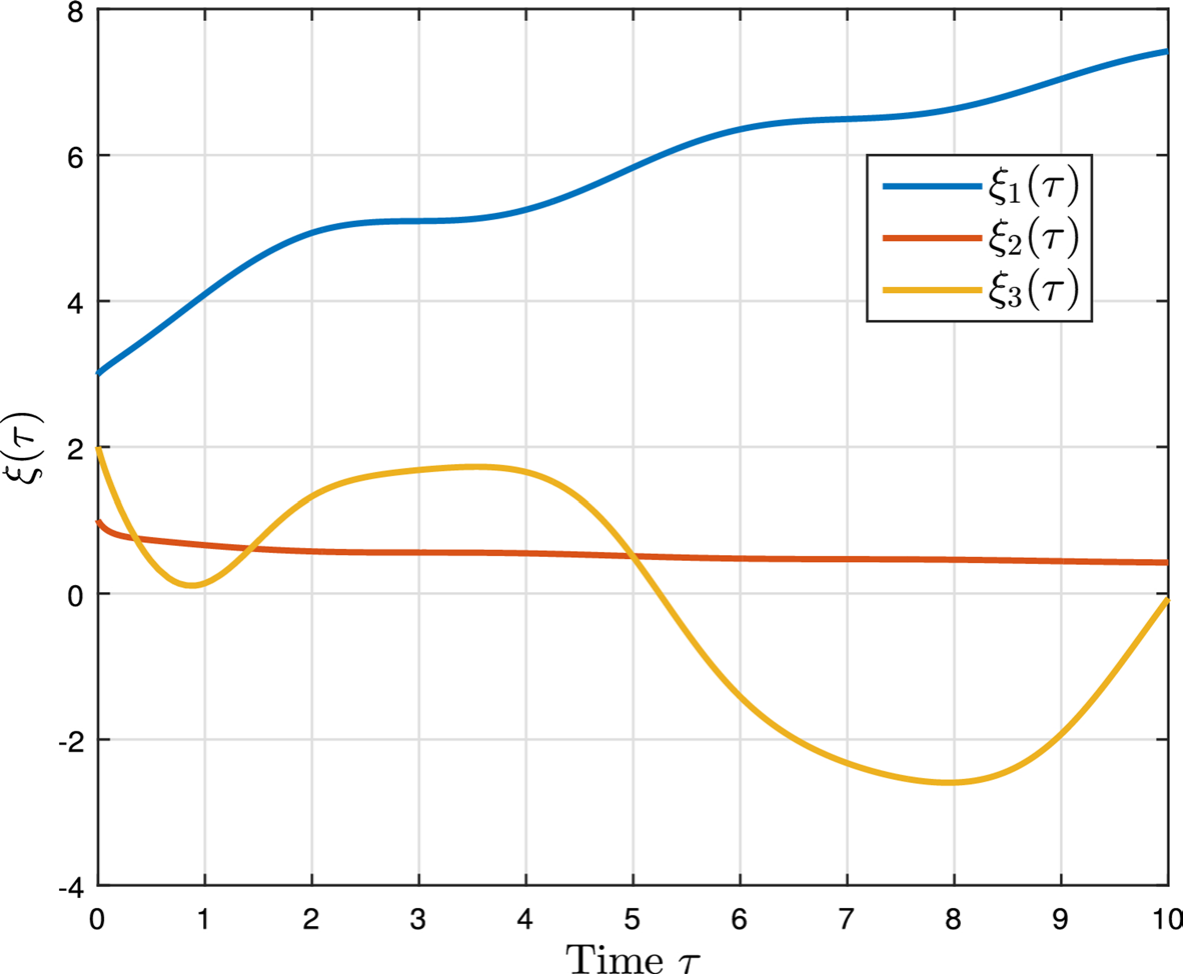
Time evolution of $\xi (\tau )$ for the CNDS in Example 2 without control $\textrm{y}(\tau )$.

Figure 6 illustrates the open-loop evolution of the state with $$\varphi (\tau )=\left[ \begin{array}{c} 3\\ 1\\ 2 \end{array}\right]$$, $\textrm{d}=2$ and $\beta =0.9$, indicating that the system is unstable.

For the performance index, we set $\sigma =0$, $\mathcal {U}=-1\; (\mathcal {U}_1=1)$, $\mathcal {V}=0$, $\mathcal {W}=\gamma ^2$, which corresponds to the $H_\infty$ performance index with level $\gamma$. All parameters that guarantee positivity of the SOS conditions in Step 2 of Algorithm 3.3 are set to $10^{-6}$, and $\alpha$ is set to 0.7. Table 1 lists the maximum allowable delay $\textrm{d}$ or different performance levels $\gamma$.

**Table 1:** Maximum delay $\textrm{d}$ for various $\gamma$.

$\gamma$	0.2	0.3	0.9
$\textrm{d}$	2.62	5.23	5.81

For $\textrm{d}=2$ and $\gamma =0.2$, we obtain the following gains:

$$\begin{aligned} & \mathcal {A}_1(\delta _1)=\left[ \begin{array}{ccc} - 2.6932&-1.64493\times 10^{-5}\,\delta _1^{2} - 65.3713&- 1.0346\times 10^{-2} \end{array} \right] ,\\ & \mathcal {A}_2(\delta _1)=\left[ \begin{array}{ccc} - 2.69245&1.51968\times 10^{-5}\delta _1^{2} - 65.48949&8.05544\times 10^{-3} \end{array} \right] ,\\ & \mathcal {A}_3(\delta _1)=\left[ \begin{array}{ccc} - 2.24550&1.58868\times 10^{-5}\delta _1^{2} - 66.32013&- 1.36836\times 10^{-3} \end{array} \right] ,\\ & \mathcal {A}_4(\delta _1)=\left[ \begin{array}{ccc} - 2.020&4.041\times 10^{-5}\,\delta _1^2 - 66.006&9\times 10^{-3} \end{array} \right] ,\\ & \mathcal {B}_1(\delta _1)=\left[ \begin{array}{cc} 0.423\,\delta _1^2 + 6.050 & 0.696\,\delta _1^2 + 3.325 \\ 0.891\,\delta _1^2 + 4.233 & 1.731\,\delta _1^2 + 7.565 \\ 0.471 & 0.001 \end{array} \right] ,\\ & \mathcal {B}_2(\delta _1)= \left[ \begin{array}{cc} 1.423\,\delta _1^2 + 6.049 & 0.696\,\delta _1^2 + 3.325 \\ 0.891\,\delta _1^2 + 4.233 & 1.731\,\delta _1^2 + 7.565 \\ - 0.016 & 0.001 \end{array} \right] ,\\ & \mathcal {B}_3(\delta _1)=\left[ \begin{array}{cc} 1.424\,\delta _1^2 + 4.957 & 0.696\,\delta _1^2 + 3.542 \\ 0.893\,\delta _1^2 + 3.883 & 1.730\,\delta _1^2 + 7.686 \\ 0.470 & 0.001 \end{array} \right] ,\\ & \mathcal {B}_4(\delta _1)=\left[ \begin{array}{cc} 1.424\,\delta _1^2 + 4.955 & 0.696\,\delta _1^2 + 3.541 \\ 0.893\,\delta _1^2 + 3.882 & 1.730\,\delta _1^2 + 7.686 \\ - 0.016 & 4.76\times 10^{-4} \end{array} \right] . \end{aligned}$$

**Fig. 7:**
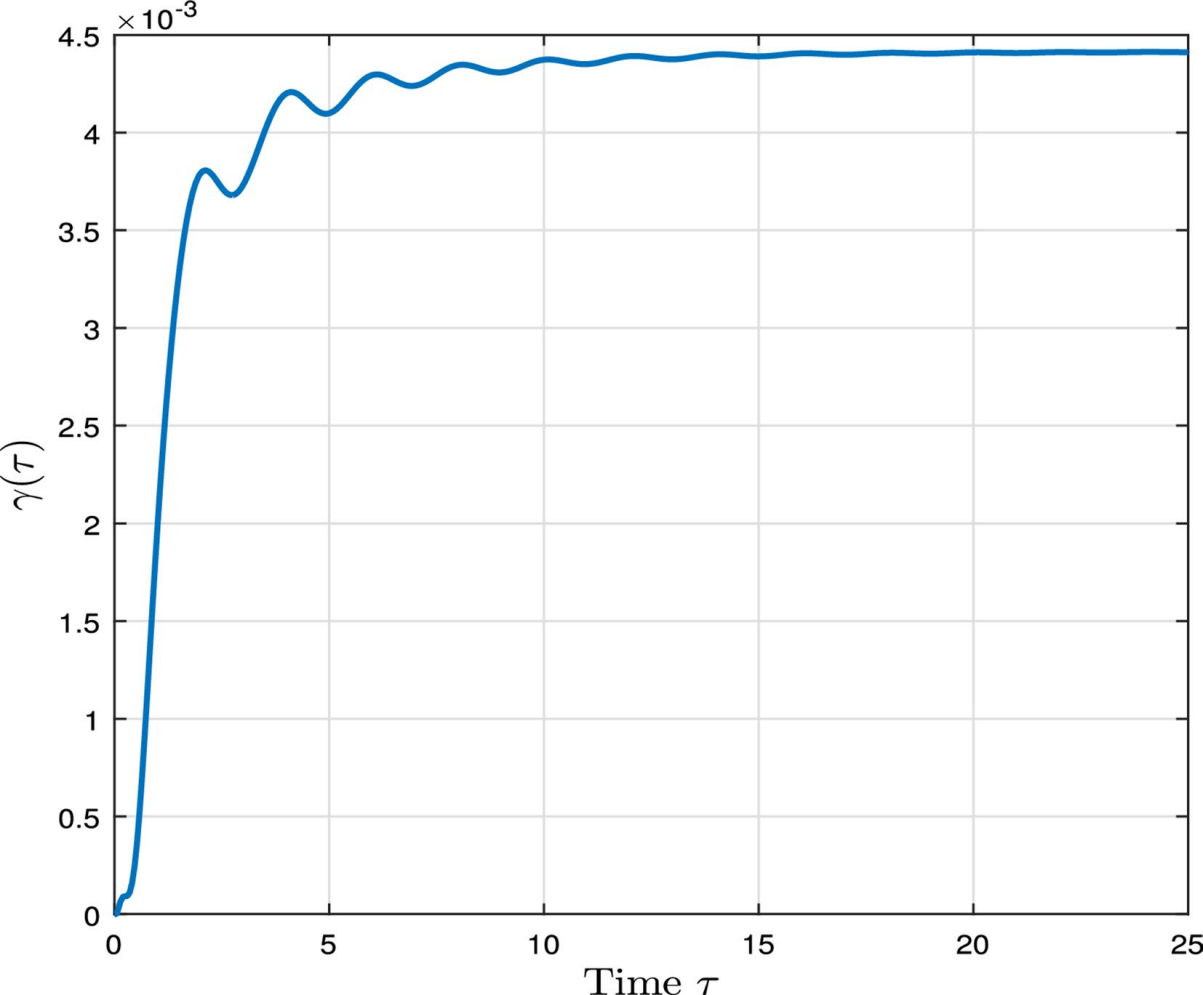
Time evolution of $\gamma (\tau )$.

Under zero initial condition, we define:

$$\gamma (\tau ) = \sqrt{ \frac{\displaystyle \int _{0}^{\tau } s^{\beta -1}\textrm{c}(s)^{T}\textrm{c}(s)ds}{\displaystyle \int _{0}^{\tau } s^{\beta -1}\textrm{p}(s)^{T}\textrm{p}(s)ds }},$$

and its evolution is shown in Fig. 7. It can be seen that $\gamma (t)$ remains below $4.5 \times 10^{-3}$, which is well under the prescribed value $\gamma = 0.2$.

**Fig. 8:**
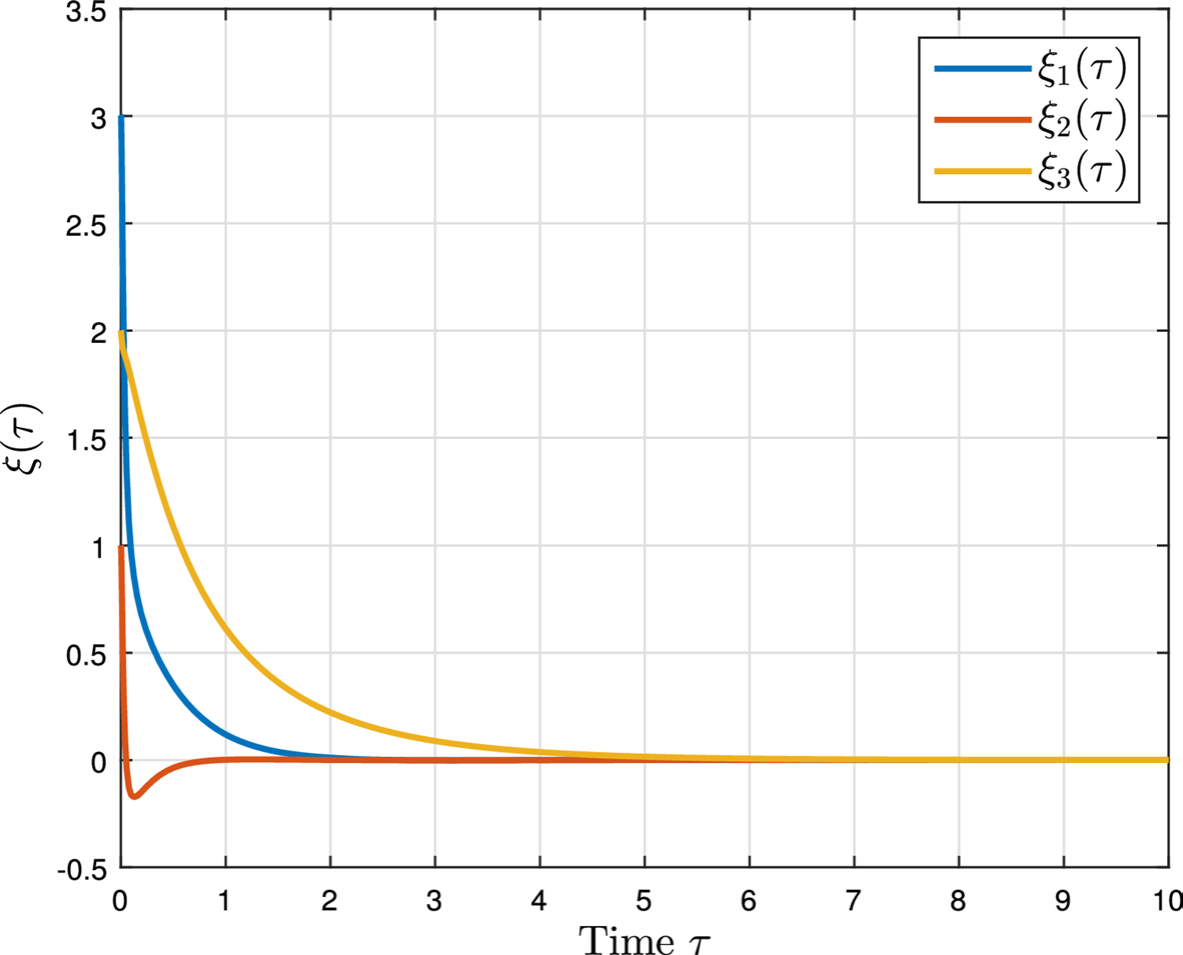
Time evolution of $\xi (\tau )$ for the CNDS with control $\textrm{y}(\tau )$ in Example 2 when $\textrm{p}(\tau )=0$.

Without any external disturbance $\textrm{p}(\tau )=0$, Fig. 8 illustrates the time evolution of $\xi (\tau )$ with control $\textrm{y}(\tau )$ under initial conditions $$\varphi (\tau )=\left[ \begin{array}{c} 3\\ 1\\ 2 \end{array}\right]$$ and $$\widehat{\varphi }(\tau )=\left[ \begin{array}{c} 0\\ 0\\ 0 \end{array}\right]$$. From this figure, it can be observed that the closed-loop fuzzy system is stable.

## Conclusion

This study investigated the exponential stabilization of conformable nonlinear systems subject to disturbance and time delay. The original nonlinear system was transformed into a PF model through sector-nonlinearity approach, yielding an accurate representation of the initial system. This generalizes the standard TSF model by allowing the consequent matrices to be polynomial rather than constant. To handle unmeasured states, the stabilizing controller is designed using a PF observer such that the resulting augmented closed-loop system is not only exponentially stable but also $(\mathcal {U},\mathcal {V},\mathcal {W})$-$\sigma$-dissipative. To derive sufficient conditions, a sum relaxation method, recently proposed for TSF models, is extended to PFM. Using a decoupling technique, the resulting conditions are equivalently reformulated as conditions that are linear in the polynomial matrices. An algorithm outlining the steps of the design procedure is presented. These steps are then illustrated through two numerical examples. Future extensions can address either modeling or control aspects. For modeling, singular PF models can be considered. In addition, approaches can be adopted to reduce the number of rules, particularly for systems with many nonlinearities. For control, extensions may include event-triggered control, self-triggered control, and fault estimation.

## Data Availability

The datasets used and/or analysed during the current study available from the corresponding author on reasonable request.
